# Evidencing the role of a conserved polar signaling channel in the activation mechanism of the μ-opioid receptor

**DOI:** 10.1016/j.csbj.2025.07.014

**Published:** 2025-07-16

**Authors:** Arijit Sarkar, Szabolcs Dvorácskó, Zoltán Lipinszki, Argha Mitra, Mária Harmati, Krisztina Buzás, Attila Borics

**Affiliations:** a Laboratory of Biomolecular Structure and Pharmacology, Institute of Biochemistry, HUN-REN Biological Research Centre, Szeged, Hungary; b Theoretical Medicine Doctoral School, Albert Szent-Györgyi Medical School, University of Szeged, Hungary; c Synthetic and Systems Biology Unit, Institute of Biochemistry, HUN-REN Biological Research Centre, Szeged, Hungary; d National Laboratory for Biotechnology, Institute of Genetics, HUN-REN Biological Research Centre, Szeged, Hungary; e Laboratory of Microscopic Image Analysis and Machine Learning, Institute of Biochemistry, HUN-REN Biological Research Centre, Szeged, Hungary; f Department of Immunology, Albert Szent-Györgyi Medical School, Faculty of Science and Informatics, University of Szeged, Hungary

**Keywords:** G protein-coupled receptors, molecular dynamics, activation mechanism, signal transduction, mutation, pharmacological assessment

## Abstract

The activity of G protein-coupled receptors has been generally linked to dynamically interconverting structural and functional states and the process of activation was proposed to be controlled by an interconnecting network of conformational switches in the transmembrane domain. However, it is yet to be uncovered how ligands with different extent of functional effect exert their actions. According to our recent hypothesis, the transmission of the external stimulus is accompanied by the shift of macroscopic polarization in the transmembrane domain, furnished by concerted movements of conserved polar amino acids and the rearrangement of polar species. Previously, we have examined the μ-opioid, β_2_-adrenergic and type 1 cannabinoid receptors by performing molecular dynamics simulations. Results revealed correlated dynamics of a polar signaling channel connecting the orthosteric binding pocket and the intracellular G protein-binding surface in all three class A receptors. In the present study, the interplay of this polar signaling channel in the activation mechanism was evidenced by systematic mutation of the channel residues of the μ-opioid receptor. Mutant receptors were analyzed utilizing molecular dynamics simulations and characterized *in vitro* by means of radioligand receptor binding and G protein stimulation assays. Apart from one exception, all mutants failed to bind the endogenous agonist endomorphin-2 and to stimulate the G_i_ protein complex. Furthermore, mutation results confirmed allosteric connection between the binding pocket and the intracellular surface. The strong association and optimal bioactive orientation of the bound agonist was found to be crucial for the initiation of correlated motions and consequent signaling.

## Highlights

• A conserved polar channel in the transmembrane domain conveys activation signal.
• Channel constituents perform concerted motions in active signaling states.
• Altering polarity of channel constituents affects ligand binding.
• Altering polarity of channel constituents affects receptor function.
• Strong allosteric connection between ligand binding pocket and intracellular surface.

## Graphical Abstract

**Figure:**
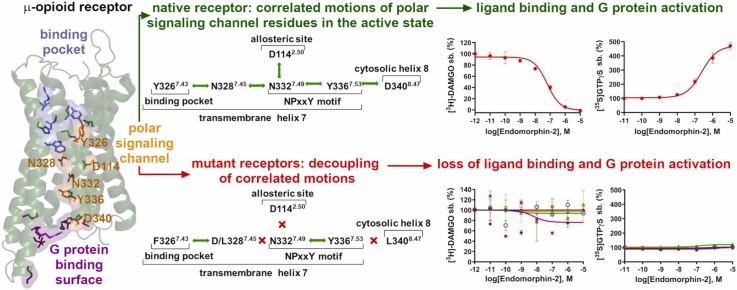

## 1 Introduction

G protein-coupled receptors (GPCRs) are one of the most coveted categories of drug targets for therapeutic interventions, evidenced by the fact that roughly one-third of all prescription drugs aim to affect receptors within this family [1], [2]. The use of certain GPCR-targeting drugs is, however, limited by undesired side effects which might result from non-selective activation of multiple GPCRs or multiple signaling pathways associated with a single receptor. A current challenge is to develop functionally selective drugs, specific to a particular signaling pathway. This is complicated by the fact that the structural mechanism of activation of these receptors as well as the ligand-receptor dynamics is still largely unclear. Only a few varieties of G proteins are involved in signaling, with the presence of ligands in far more excess in comparison, which suggests that GPCR activation may follow a general mechanism. This information is critical in designing drugs to selectively target specific signaling pathways, thereby reducing side effects and improving therapeutic outcomes. According to the common theory of GPCR activation, the receptor undergoes conformational changes upon binding to agonists that activate downstream signaling pathways. These pathways include the G protein-coupled pathway, which involves the activation of G proteins and subsequent regulation of secondary messengers such as cyclic adenosine monophosphate (cAMP) [3] as well as other intracellular signaling pathways, such as the mitogen-activated protein kinase (MAPK) [4] and phospholipase C (PLC) pathways [5].

Structurally, GPCRs are transmembrane proteins which consist of a conserved domain of seven transmembrane helices (TM domain) connected by intra and extracellular loops, an extracellular N-terminal domain, and an intracellular C-terminal domain. In the recent years, there has been significant research interest in unraveling the structure of GPCRs in high spatial resolution, leading to the advancement of cutting-edge techniques. From the traditional x-ray and lipidic cubic phase (LCP) crystallography to emerging methodologies such as single-particle cryo-electron microscopy (cryo-EM) and x-ray free-electron laser (XFEL) protein crystallography, a plethora of state-of-the-art techniques have been developed for this purpose. Such developments have enabled researchers to obtain high-resolution structures of GPCRs [6]. The knowledge of the process of transitioning from the inactive state to the active signaling state of GPCRs has become increasingly complex as novel discoveries continue to emerge each year. Upon activation of GPCRs, there is a remarkable level of structural conservation in the intracellular changes that occur, indicating a common evolutionary origin for the activation mechanism across most GPCRs. The above studies as well as landmark molecular dynamics simulation results indicated that the activation process of class A GPCRs is accompanied by the displacement of the 6th transmembrane helix (TM6), resulting in the creation of a cavity on the receptor's intracellular face that can accommodate the C-terminus of the G protein α-subunit. Additionally, transmembrane helix 5 (TM5) also rotates away from the receptor, further enlarging the cavity for G protein binding. The dynamic nature of such receptors can be showcased by the suggestion that GPCRs can exist in dynamically interconverting active, inactive and intermediate structural states, even in the absence of ligands. The population of such states relies upon ligand binding and orientation, physico-chemical properties of the ligand, interacting intracellular signaling proteins and lipid membrane composition, among other factors [7], [8], [9], [10], [11], [12]. Recent studies have shed light on the potential role of several other variables on activation. Key conserved functional motifs, including E/DRY, NPxxY, and CWxP, are recognized to play a role in the activation process of class A GPCRs. Additionally, when TM helices undergo reorganization, it is usually accompanied by the synchronized interplay between the mentioned conserved motifs, and a network of water molecules present in the inner cavities of the TM domain. The continuous polar pathway, created by this network, functions as a linkage between the ligand binding pocket and the intracellular G protein binding surface. It has been suggested as the fundamental pathway of receptor activation. Furthermore, the elevated levels of Na^+^ leading to inhibition of agonist-induced activation of opioid receptors and related GPCRs, due to a preserved allosteric Na^+^ binding site was revealed [13], [14], [15], [16], [17], [18], [19], [20].

Results of a recent, extensive MD simulation study involving the μ-opioid receptor (MOP), a class A GPCR, in active and inactive states showed highly correlated internal motions among the side chains of the conserved functional motifs; the orthosteric and allosteric binding pockets, the NPxxY motif, and the cytosolic helix (H8), mentioned earlier, which form a polar signaling channel connecting the ligand binding pocket to the intracellular surface. These ordered synchronous motions were exclusively observed in the active state MOP when bound to the G_i_ protein and not in the inactive state, or when bound to the β-arrestin-2 complex, indicating that these motions could be associated with G protein-mediated signaling [25]. Follow-up MD simulation studies involving two different class A GPCRs corroborated the above findings, suggesting the existence of a generalized activation pathway for class A GPCRs [26], [27]. To address the uncertainties around biased signaling an auxiliary hypothesis was formulated that states, that receptor activation is accompanied by a shift of electrostatic balance in a central duct of the TM domain. The electrostatic balance in the orthosteric pocket is perturbed upon ligand binding which perturbation is then propagated to the intracellular surface of the receptor through the synchronous movements of conserved polar amino acid side chains, assisted by water molecules, leading to G protein dissociation and subsequent downstream signaling. The hypothesis could be supported by the fact that mutations affecting the polarity of functional motifs in class A GPCRs can have a significant impact on their function as well as their ligand binding affinity [17], [24], [28], [29], [30], [31], [32], [33], [34], [35], [36], [37]. Additionally, Na^+^ binding in the allosteric site has been shown to inhibit the activation of many class A GPCRs, further suggesting the importance of charge balance and polarity changes in receptor signaling [17], [19].

In this research, we investigated the mechanisms underlying MOP activation and observed the effect of altering the polarity of the proposed signaling channel on ligand binding and the signaling activity of the receptor. Multiple mutants of the MOP receptor were designed and generated, in which the polarity of signaling channel constituents was modified to different extents. The effects of mutations were examined via simulation and experimental approaches.

## 2 Methods

### 2.1 Design of mutant MOP receptors

**Fig. 1:**
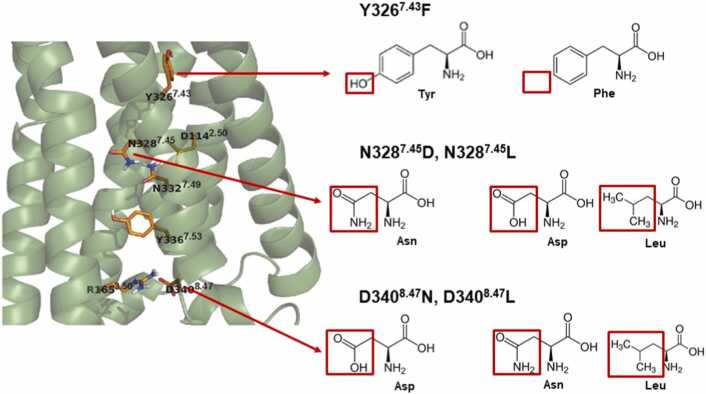
Selected mutations aimed at altering the polarity of the proposed signaling channel of the MOP.

Following up on our study involving MOP [25], point mutations were introduced in the participants of the polar signaling channel, namely Y326^7.43^F, N328^7.45^D, N328^7.45^L, D340^8.47^N, and D340^8.47^L. These residues belong to the orthosteric pocket, the allosteric Na^+^ anchoring site, and the intracellular G protein coupling interface, respectively (Fig. 1). In previous studies involving different GPCRs, mutations of the elements of the polar signaling channel led to impaired G protein signaling or increased constitutional activity [17], [28], [29], [30], [31], [32], [33], [34], [35], [36], [37]. The choice of the mutants for our investigation was based on their positions, and lack of extensive studies for most of the residues concerning MOP.

### 2.2 Preparation of simulation systems

The cryo-EM structure of the active state MOP-G_i_ protein complex (PDB code: 6DDE) [23], was obtained from the Brookhaven Protein Data Bank (http://www.rcsb.org). Additionally, separate coordinates of the G_i_ protein heterotrimeric complex and GDP (PDB code: 1GP2) [38] were used as essential components in the simulations. GTP was generated in CHARMM-GUI [39] and replaced GDP from the G_i_ complex. The G_i_ protein heterotrimeric complex of 1GP2 was aligned to the G protein coordinates of 6DDE. The crystallization chaperones and fusion proteins were excluded from the starting structures. The protonation states of ionizable amino acid side chains were assigned using the PropKa server [40]. Single point mutants of the MOP were generated virtually, using the mutagenesis function of Pymol 2.4.0., keeping focus on the effect of alteration of the polarity of the established signaling channel. Specifically, Y326^7.43^ in the ligand binding pocket was mutated to F; N328^7.45^, a component of the allosteric Na^+^ binding pocket, was mutated to D and L, and D340^8.47^, a part of the intracellular G protein coupling interface, was mutated to N and L (Fig. 1).

The sequences of the murine MOP (UniProtKB-P42866-OPRM1) were retrieved from the UniProt database (http://www.uniprot.org). 10 ns folding simulations were performed to reconstruct the flexible N- and C-terminal domains which are missing from their experimental structures. The simulation utilized the GROMACS 2018.3 program package [41], the AMBER ff99SB-ILDN NMR force field [42], and the GB/SA implicit solvation model [43], in accordance with a previously established protocol [25]. Any additional missing, modified, or mutated residues within the structure of the receptor were restored or corrected using the Swiss-PdbViewer program ver. 4.10 [44]. Throughout MD simulations, the system temperature was set to 310 K, and v-rescale algorithm was used for its regulation [45]. Ten parallel simulations were conducted for both the terminal domains, and the resulting folded structures were assessed and chosen based on their compactness and the accessibility of post-translational modification (PTM) and TM region attachment sites. The suitably folded terminal domains were then appended manually to the TM region using Pymol 2.4.0.

The PTMs in the receptor structures, including glycosylation of the N-terminal domain, were incorporated using the CHARMM-GUI web-based platform [39]. The glycosylation involved a common core structure (Manα1–3 (Manα1–6) Manβ1–4GlcNAcβ1–4GlcNAcβ1–N) along with sialic acid (N-acetylneuraminic acid). Phosphorylation of serine and threonine residues in the C-terminal domain, along with palmitoylation of a cysteine residue in the 3rd intracellular loop (ICL3), were also introduced. A single disulfide bridge between two cysteine residues, which play a critical role in stabilizing the structure of the protein was also added. The sites of the PTMs are noted in Table S1.

The structure of endomorphin-2 (EM2), an endogenous peptide agonist of the MOP [46] was constructed manually using Pymol 2.4.0. Since these simulation systems were built prior to the publication of the cryo-EM structure of MOP bound endomorphin-1 (EM1), a peptide agonist analogous to EM2 [24], molecular docking was utilized to predict the binding site and orientation of EM2 in MOP. The Autodock 4.2 software [47], employing the Lamarckian genetic algorithm, was used for the flexible docking of EM2 to the active state MOP experimental structure, to verify its orthosteric binding site. The docking was conducted within an 8.0 nm × 8.0 nm × 8.0 nm grid volume, ensuring comprehensive coverage of the receptor surface accessible from the extracellular side, including the ligand binding pocket. All φ, ψ, and χ^1^ ligand torsions, as well as receptor side chains in contact with the bound ligand (D147^3.32^, Y148^3.33^, M151^3.36^, K233^5.39^, W293^6.48^, I296^6.51^, H297^6.52^, V300^6.55^, I322^7.39^, Y326^7.43^), were kept flexible during the docking procedure. The grid points were spaced at intervals of 0.0375 nm, and a total of 1000 dockings were performed. The resulting ligand-receptor complexes underwent clustering and ranking based on their corresponding binding free energies. The lowest energy bound state, indicating the presence of the specific ligand-receptor interactions observed in the cryo-EM structure of MOP with peptide agonist DAMGO, was selected verifying the optimal localization of EM2 in the pocket. The orientation of EM2 was meticulously scrutinized to adhere to its bioactive conformation, drawing insights from both the experimental structure and our studies [10], [25]. The cryo-EM structure of the MOP - EM1 complex published later have validated the orientation of EM2 used in our studies.

To emulate their native environment, membrane bilayers with caveolar compositions were constructed based on lipidomic data [48], also validated by our earlier studies [25], [26], [27]. The membrane builder tool of CHARMM-GUI was used to build the membrane system with the lipid components being parameterized according to CHARMM36m parameters [39]. The receptor complexes were incorporated into a resultant asymmetric multicomponent, raft-like membrane systems composed of cholesterol (CHL ∼32.8 %), 1-palmitoyl-2-oleoyl-glycero-3- phosphatidylcholine (POPC ∼14.9 %), 1-palmitoyl-2-oleoyl-sn-glycero-3-phosphatidylethanol- amine (POPE ∼27.8 %), 1-palmitoyl-2-oleoyl-sn-glycero-3-phosphatidyl-L-serine (POPS ∼3.6 %), 1- palmitoyl-2-oleoyl-sn-glycero-3-phosphatidylinositol (POPI2 ∼6 %), palmitoyl-sphingomyelin (PSM ∼9.9 %) and monosialodihexosylganglioside (GM3 ∼5 %). According to recent literature data, the asymmetric composition of the lower and upper leaflets was determined [49]. The systems were explicitly solvated with TIP3P water molecules in a hexagonal-shaped periodic box. To achieve physiological ionic strength, Na^+^ and Cl^-^ ions (0.15 mM) were added and the net charge of the system was set to neutral. The coordinates and topologies of the systems were generated in GROMACS format, with CHARMM36m all-atom force field parameters assigned to the generated components [50]. Altogether, a total of 18 independent 1 µs simulations were set up in the workflow for the MOP complexes.

### 2.3 MD simulations

Following the building of a caveolar membrane around the receptor complexes, the GROMACS 2018.3 program package [41] was utilized to perform thorough energy minimization and subsequent equilibration of the whole system, closely following the protocol established in our previous works [25], [26], [27]. The simulation systems were subjected to an initial 5000 steps of steepest descent, and subsequent 5000 steps of conjugate gradient minimization. The convergence criterion for both steps was set at 1000 kJ mol^−1^ nm^−1^. Using a six-step protocol supplied by CHARMM-GUI, the equilibration process involved two consecutive MD simulations at 303.15 K in the canonical (NVT) ensemble, followed by four additional simulations in the isobaric-isothermal (NPT) conditions at the same temperature and 1 bar pressure. Positional restraints were imposed on the heavy atoms of the proteins and membrane constituents and gradually decreased throughout the equilibration protocol. The initial three equilibration MD runs were 25 ps in duration and conducted with 1 fs time steps. The subsequent two runs continued for 100 ps with 2 fs time steps, and the final equilibration step was extended to 50 ns and was executed in 2 fs time steps, similar to the approach described previously [25], [26], [27]. The correct bond lengths were maintained using the LINCS algorithm, temperature was regulated using the v-rescale algorithm [45] with a coupling constant of 1 ps, and pressure control was achieved using the c-rescale barostat [51]. The pressure coupling method had a coupling constant of 5 ps and isothermal compressibility of 4.5 × 10^−5^ bar^−1^. Energy contributions from long-range electrostatic interactions were calculated using the particle mesh Ewald (PME) summation method, and van der Waals interactions were computed with a twin-range cutoff. 1.2 nm was set as the cut-off value, as described earlier [25], [26], [27].

Executing GROMACS 2018.3, unrestrained production simulations for the wild type receptor and its mutant systems were carried out in the NPT ensemble at 310 K, employing parameters akin to those mentioned above. Each production simulation extended for 1 µs. System coordinates were recorded every 5000 steps, yielding 100,000 snapshots per trajectory. Simulations of the active state wild type receptor as control were performed as well as simulations of its mutants mentioned earlier, complexed with the heterotrimeric G_i_ protein. These simulations were performed in 3 replicates per system, in the presence of the endogenous agonist EM2, oriented in its bioactive conformation (see above).

### 2.4 MD trajectory analysis

The analysis suite of the GROMACS 2018.3 package was used for the analysis of our output MD trajectories as outlined in our previous studies [25], [26], [27]. The radius of gyration of terminal domains, along with the measurement of the minimum distance between them was performed, to test the integrity of the simulation system, using *gmx gyrate* and *gmx mindist*, respectively. Potential artifacts caused by the proximity of the flexible terminal domains and their periodic replicas were also evaluated via these methods. The minimum distance between Na^+^ from the allosteric site was also assessed to report its penetration and localization. Root mean square deviation (RMSD, *gmx rms*) and root mean square fluctuation (RMSF, *gmx rmsf*) of the protein backbone atoms was measured to investigate the structural deviation of domains with respect to the starting experimental structures. Specific interactions between conserved microswitches were calculated by analyzing the presence of H-bonds as well as salt bridges between the residues, utilizing *gmx hbond*, *gmx distance* and *gmx angle*. For the H-bond, the donor-acceptor distance and angle cutoff were set to 0.35 nm and 30.0 degrees or below, respectively; whereas for the salt bridge calculations, the distance and angle cut-off between the acidic and basic moieties were set to 0.4 nm and 90.0 degrees. The secondary structures of receptor domains were assigned and monitored during simulations using the DSSP (Define Secondary Structure of Proteins) method [52]. Side chain rotamer conformations were also measured using *gmx chi* to determine the signaling state of the receptor. The correlation of side chain motions was evaluated using generalized cross-correlation matrix (GCCM) analyses based on linear mutual information (MI), using the *g_correlation* utility of the GROMACS 3.3 program package, following the methodology used in our previous work [25], [26], [27]. Briefly, correlation matrices were converted to heat maps using the GIMP ver. 2.10.30 software, with red intensity levels corresponding to MI values greater than 0.63. Principal component analysis (PCA) and the generation of Gibbs free energy landscapes was carried out using *gmx covar*, *gmx anaeig* and *gmx sham*. Clustering was performed using the *gmx cluster* utility and the gromos method [53], comparing backbone atoms of the TM domain with 1.0 Å and 1.5 Å RMSD cutoff. MMPBSA calculations have been performed using the gmx_MMPBSA utility [54], [55]. Visualization of molecular structures was carried out using Pymol 2.4.0, and graphs were created using the Xmgrace ver 5.1.25 program.

### 2.5 DNA constructs

The full-length complementary DNA (cDNA) of the mouse MOP receptor (GenBank #: AB047546.1), which includes the coding sequence (CDS) as well as the 5’ and 3’ untranslated regions (UTRs), was amplified using polymerase chain reaction (PCR) with Phusion Plus DNA polymerase (Thermo Fisher Scientific, Waltham, MA, USA, #F630). Genomic DNA extracted from the CHO-MOP cell line [56] (kindly provided by András Váradi, Memorial Sloan-Kettering Cancer Center, New York, United States) served as the template. The PCR product was blunt-end cloned into the pJET2.1 plasmid according to the manufacturer’s instructions (Thermo Fisher Scientific, #K1231). Verification of the wild-type MOP (MOP WT) was confirmed by DNA sequencing. Modified MOP constructs, each with a specific amino acid substitution (Y326^7.43^F, N328^7.45^D, N328^7.45^L, D340^8.47^N, or D340^8.47^L), were generated using the QuickChange II XL Site-Directed Mutagenesis Kit (Agilent Technologies, Santa Clara, CA, USA, # 200523). The cDNAs of these MOP variants were then PCR-amplified from the pJET2.1 plasmids and inserted into the *Bam*HI site of the pTRE2hyg mammalian expression vector (Clontech, Palo Alto, CA, USA, #6255–1) using In-Fusion cloning (Takara Bio, Shiga, Japan, #639648). All constructs were confirmed by DNA sequencing before transfection. The oligonucleotide primers used are listed in Table S2.

### 2.6 Generation of stably transfected CHO-K1 Tet-On cell lines

To generate stable CHO-K1 Tet-On cell lines expressing MOP derivatives, purified plasmid DNA containing either WT or mutated forms of MOP cDNA in the pTRE2hyg vector was transfected into CHO-K1 Tet-On cells (Clontech, #C3021–1) using Torpedo-DNA transfection reagent (Ibidi, Gräfelfing, Germany, #60611) following the manufacturer’s guidelines, as previously described [57]. An empty pTRE2hyg vector was used to establish the control cell line. Selection of stably transfected cell lines began 24 h post-transfection in low glucose-containing DMEM medium (Biosera, Nuaille, France, #LM-D1102), supplemented with 10 % tetracycline-free fetal bovine serum (Biosera, #FB-1001T), 1× Penicillin-Streptomycin (Biosera, #XC-A4122), 2 mM stable glutamine (Biosera XC-T1755), 100 µg/mL G418 sulfate (Corning, NY, USA, #61–234), and 250 µg/mL Hygromycin B (Corning, #30–240-CR). The selection process continued for 2–3 weeks with regular medium changes until distinct colonies were visible. Cells were cultured in 6 cm or 10 cm tissue culture dishes in a 37 °C humidified incubator with 5 % CO_2_. Transgene expression was induced by treating the cells with 1.5 µg/mL doxycycline (Clontech, cat# 631311) for 24 h.

### 2.7 Membrane preparation

Membranes of CHO-K1 cells stably expressing the wild type and mutant MOP were prepared from 4 monolayer cell cultures in 175 cm^2^ flasks. Cells were rinsed with DPBS 3 times and dissociated with 0.5 mM EDTA (Merck Millipore, Burlington, MA, USA, #324503) for 8–10 min at room temperature. Then, the cells were washed with DPBS (Lonza, # 17–515 F) via centrifugation at 1500× g for 10 min at 4 °C. Cell pellet was resuspended in 50 mM Tris–HCl (pH 7.4), frozen at -80 °C for 1 h and homogenized with Dounce homogenizer after thawing. Homogenate was ultracentrifuged at 100,000× g for 30 min at 4 °C. Then, the pellet was resuspended in 50 mM Tris–HCl (pH 7.4) and stored in aliquots at -80 °C. Protein concentration of the membrane preparations was measured with the Pierce™ BCA Protein Assay Kit (Thermo Fisher Scientific, #23225) based on the manufacturer’s instructions.

### 2.8 Radioligand competition binding assay

Binding assays were carried out following a previously published protocol [58], at 25 °C for a duration of 60 min using a 50 mM Tris–HCl buffer (pH 7.4) in glass tubes. Each assay had a total volume of 1 mL and contained 0.5 mg/mL of membrane protein. For competition binding assays, cell membranes were exposed to 2 nM of [^3^H]DAMGO (K_d_: 0.5 nM) along with increasing concentrations (10^−12^ - 10^−5^ M) of synthetic, unlabeled EM2 (generously provided by Géza Tóth from the Institute of Biochemistry, HUN-REN Biological Research Centre, Szeged, Hungary) [59]. Non-specific binding was evaluated using 10 μM naloxone. Following incubation, samples were diluted with an ice-cold wash buffer (50 mM Tris–HCl, pH 7.4) and then subjected to multiple washes. The samples were rapidly filtered through Whatman GF/C glass fiber filters (Whatman Ltd., Maidstone, UK) using a 24-well Brandel Cell Harvester (Gaithersburg, MD, USA). The filters were subsequently air-dried, immersed in Ultima Gold MV scintillation cocktail, and the radioactivity was quantified with a TRI-CARB 2100TR liquid scintillation analyzer (Packard, Perkin Elmer, Waltham, MA, USA).

### 2.9 Ligand stimulated [^35^S]GTPγS binding assay

The ligand-stimulated [^35^S]GTPγS binding assays were performed as outlined previously [58]. Briefly, cell membranes (30 μg protein/tube) were prepared and incubated with 0.05 nM of [^35^S]GTPγS (PerkinElmer) and varying concentrations (10^−11^ - 10^−5^ M) of unlabeled EM2 in a buffer containing 30 μM GDP, 100 mM NaCl, 3 mM MgCl_2_, and 1 mM EGTA in 50 mM Tris–HCl buffer (pH 7.4). The incubation was performed at 30 °C for 60 min. Basal [^35^S]GTPγS binding was recorded in the absence of ligand and was used as a reference value (100 %). Non-specific binding was determined by the addition of 10 μM unlabeled GTPγS, which was then subtracted from the total binding. The processes of incubation, filtration, and measurement of radioactivity were executed as described above.

### 2.10 Data analysis

The calculation of inhibitory constants (K_i_) during competition binding assays was conducted by analyzing the inflection points of the displacement curves through nonlinear least-squares curve fitting, as previously reported [60]. These constants were determined using the Cheng-Prusoff equation: K_i_ = EC_50_/(1+ [ligand]/K_d_). For the [^35^S]GTPγS binding assays, data were reported as the percentage increase in specific [^35^S]GTPγS binding compared to basal levels. Each assay was performed in triplicate, and potency (EC_50_) and efficacy (E_max_) were assessed using sigmoid dose-response curve fitting methods, consistent with the procedures detailed in our earlier publication [60]. Statistical analysis of E_max_ and EC_50_ values was carried out using one-way ANOVA followed by Tukey's multiple comparison test (^****^P < 0.0001) and unpaired Student's *t*-test. Furthermore, comparisons of [^35^S]GTPγS binding values for EM2, in the presence or absence of 1 and 10 μM naloxone, and RMSD values obtained from MD simulation results were performed using one-way ANOVA with subsequent Tukey's test (^****^P < 0.0001, ^**^P < 0.01, *P < 0.05). All data analyses and curve fitting were executed using GraphPad Prism 9.0 Software (San Diego, CA, USA), as described earlier [60].

## 3 Results and discussion

### 3.1 Simulation system integrity

Building upon the methodology employed in our previous studies [25], [26], [27], we adopted a similar approach in constructing the simulation systems. Particular attention was given to incorporating both the N- and C-terminal domains of the receptor while constructing the simulation systems, to achieve a realistic approximation of the native structure. This inclusion was intended for accounting for the drag caused by the mass of these domains and its subsequent impact on the dynamics of the transmembrane helices whose significance in the activation of the receptor has been greatly emphasized [7], [15], [20]. Consequently, their accurate representation was deemed essential in the simulation systems developed for this study.

During the course of simulations, a notable observation was the frequent occurrence of partial unfolding in the N- and C-terminal domains, evidenced by the changes in the radii of gyration over time (Figure S1). It was consistently observed, however, that the minimum distance between the terminal domains remained above 4.0 nm, and the periodic box size was large enough to exclude any potential artifacts arising from artificial contacts or overlaps between neighboring periodic images of the domains (Figure S2).

No dissociation of EM2 from the binding pocket was observed in any set of the simulations. The ligand roughly maintained its initial position throughout the simulations, similar to that observed for the superagonist peptide DAMGO in the cryo-EM structure with regard to the spatial arrangement of pharmacophores (Figure S3) [23]. Interestingly, the χ^1^ torsional angle of F^3^ of EM2 adopted *gauche-* conformation dominantly, for most systems, in agreement with a previous proposal for the bioactive conformation of opioid peptides (Table S3) [61].

### 3.2 Functional dynamics

#### 3.2.1 Allosteric Na^+^ binding

**Fig. 2:**
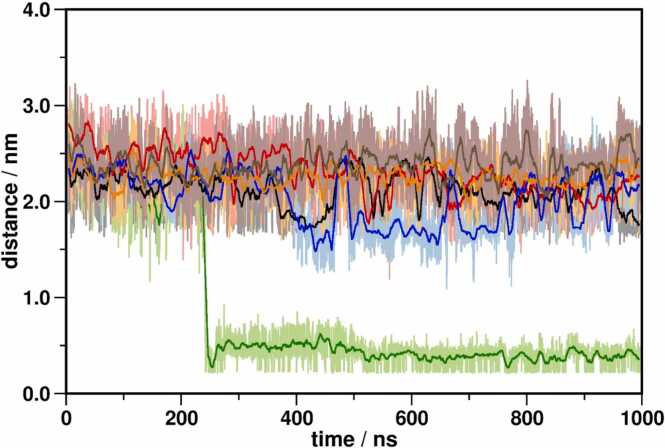
The minimum distance between Na^+^ ions and D114^2.50^ of the allosteric binding pocket in the first replica of simulations of each receptor system. Black: wild type MOP; red: Y326^7.43^F; green: N328^7.45^D; blue: N328^7.45^L; orange: D340^8.47^N; brown: D340^8.47^L.

The role of Na^+^ as a negative allosteric modulator for class A GPCRs, along with its allosteric binding site has been well documented, especially in ligand-free states of the receptor [62], [63], [64]. In line with the allosteric stabilization of the inactive state by Na^+^, the active-state structure of the receptors typically does not exhibit the presence of a Na^+^ ion. The allosteric Na^+^ binding site, as suggested by previous simulations, is only accessible through the orthosteric pocket [25], [26], [27]. In the case of mutant MOP complexes, only the mutant N328^7.45^D showed Na^+^ penetration. (Fig. 2, Table 1) In contrast with earlier observations, Na^+^ inserted into the N328^7.45^D mutant MOP receptor in spite of the bound ligand, suggesting increased dynamics and lower stability of the receptor ligand complex compared to the other systems. However, such potential destabilization was not corroborated by ligand displacement analysis. (Figure S3) Intracellular access of Na^+^ ions through the TM domain is conceivably obstructed by intracellular signalling proteins, as the entrance of Na^+^ from the cytosolic side did not occur in any of the systems.

**Table 1:** Frequency of Na^+^ ions present in the allosteric binding pocket.

**Na**^**+**^**within 0.4 nm of D114**^**2.50**^	**WT**	**Y326**^**7.43**^F*	**N328**^**7.45**^**D**	**N328**^**7.45**^**L**	**D340**^**8.47**^**N**	**D340**^**8.47**^**L**
**replica 1**	0.0	0.0	25.50	0.0	0.0	0.0
**replica 2**	0.0	0.0	35.49	0.0	0.0	0.0
**replica 3**	0.0	0.0	22.62	0.0	0.0	0.0

* Ballesteros-Weinstein numbering is shown in superscript

#### 3.2.2 Transmembrane helix dynamics

**Fig. 3:**
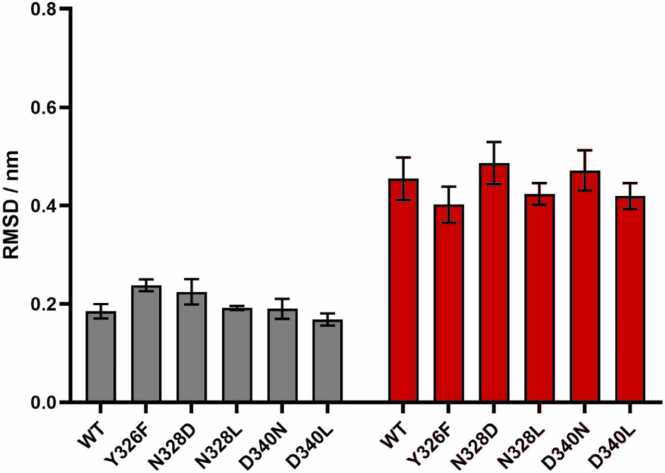
Disposition of TM6 helix backbone atoms of wild type (WT) and mutant receptors relative to active (grey) and inactive (red) experimental reference structures. Bars represent the mean ± SEM of three independent replicates (n = 3). Statistical significance was assessed using two-way ANOVA followed by Tukey’s multiple comparisons test. No statistically significant differences were observed among the groups (p > 0.05).

The disposition of TM6 in the transmembrane domain serves as a general indicator of the activation state of class A GPCRs, also referred to as the ‘signature’ conformational switch. In all simulation setups, analysis of the dynamics of TM6 helix indicated small to moderate displacements from the corresponding starting structures (Fig. 3). TM6 maintained active-like/intermediate positions in all systems throughout the simulations and no complete transitions to inactive receptor conformations were observed. This finding aligns with previous simulation results where larger displacements of TM6 were only observed in the ligand free system, or at much longer timescales [8], [25], [26], [27].

**Fig. 4:**
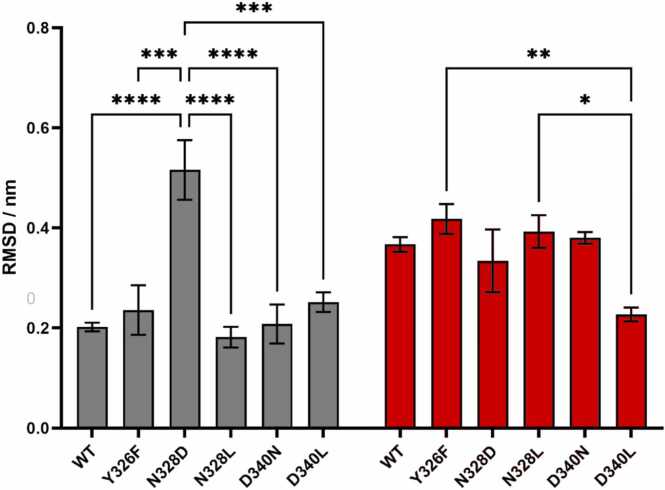
Disposition of the NPxxY motif backbone atoms of wild type (WT) and mutant receptors relative to active (grey) and inactive (red) experimental reference structures. Bars represent the mean ± SEM of three independent replicates (n = 3). Statistical significance was assessed using two-way ANOVA followed by Tukey’s multiple comparisons test. Significance is indicated as follows: ^****^p < 0.0001, ^***^p < 0.001, ^**^p < 0.01, * *p* < 0.05.

The conserved NPxxY motif, located at the junction of TM7 and H8, is implicated in stabilizing intermediate conformations in ligand-free class A GPCRs [8], [21]. These intermediate conformations are crucial for facilitating the insertion of G proteins. Notably, significant displacements of the conserved NPxxY motif were considered a contributing factor to the mobility of the polar channel segment closer to the intracellular surface, associated with the activation of the β_2_AR and type 1 cannabinoid (CB1) receptors [36], [37]. The wild type MOP and its mutants generally exhibited minimal deviations of the NPxxY motif, with the exception of the N328^7.45^D mutant. The N328^7.45^D mutant displayed a substantial displacement of the motif from its initial active state position (Fig. 4). This noteworthy observation, coupled with the reported Na^+^ penetration, suggests the profound impact that a single mutation can have on the receptor, influencing the polarity of the signaling channel preceding the NPxxY motif.

**Fig. 5:**
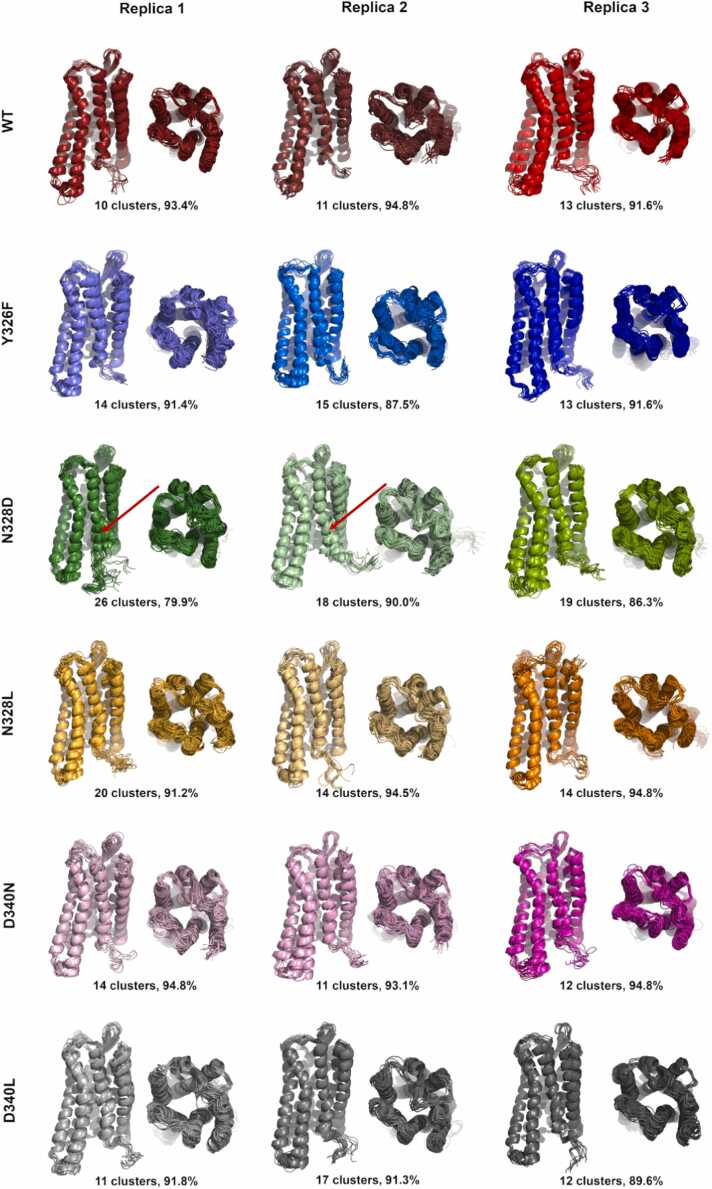
Overlaid middle structures of the clusters with population exceeding 1.0 % of the total structural ensemble of each system. The text below the individual images indicates the number of clusters with population exceeding 1.0 % of the total structural ensemble and the sum of the population of these clusters. Red arrows denote considerable destabilization of TM7.

Principal component analysis (PCA) of the TM domain dynamics indicated multiple stable and accessible conformational states in case of all receptor systems and simulation replicas. (Figure S4). However, results of cluster analysis, where the similarity cutoff of backbone RMSD was set to 1.0 Å, a value below common experimental resolution, revealed that the different conformational states are in fact highly similar. (Fig. 5, Table S4) Moderate internal dynamics was observed for the intracellular ends of TM5, TM6 and TM7 compared to TM1–4, of which structures remained broadly uniform during the simulations, in agreement with earlier observations, regardless of the receptor type [7], [8], [9], [10]. The largest degree of destabilization was observed for the intracellular end of TM7, including the NPxxY motif, in the case of the N328^7.45^D mutant (indicated with red arrows in Fig. 5). RMSF analysis of TM residues have confirmed that TM6 (residues 268–306) and TM7 (residues 311–340) are slightly more mobile than the other TM helices. (Figure S5) TM4 (residues 180–206) has also shown moderate dynamics, whereas clustering did not indicate considerably different structural states with regard to this helix. The lifetime and/or frequency of these structural states could be significantly lower than those emerging from the internal motions of TM6 and TM7.

#### 3.2.3 Intramolecular interactions

The results of MD trajectory analysis for specific intramolecular salt bridges and hydrogen bonds, previously recognized as pivotal molecular switches for class A GPCR activation, are summarized in Table 2 [21], [22], [28], [65], [66], [67], [68]. No significant or relevant differences were found with regard to the frequencies of these intramolecular interactions. Experimental data suggests that the salt bridge and hydrogen bond between the D^3.49^ and R^3.50^ residues of the DRY motif remain absent in most active state GPCRs. Consistent with the data, MOP and its mutants, where we dealt with just the active states, these interactions were primarily absent as expected.

**Table 2:** Frequency of specific intramolecular salt bridges and H-bonds expressed as percentages of the total conformational ensemble, generated by MD simulations. Values represent mean ± SEM from three independent replicates (n = 3).

**Interactions**	Residues involved*	**WT**	**Y326**^**7.43**^**F**	**N328**^**7.45**^**D**	**N328**^**7.45**^**L**	**D340**^**8.47**^**N**	**D340**^**8.47**^**L**
**Salt bridges**							
**DRY – H8**	R165^3.50^; D340^8.47^	33.5 ± 4.2	25.3 ± 20.6	2.1 ± 1.1	56.9 ± 27.9	N/A	N/A
**intra-DRY**	D164^3.49^; R165^3.50^	8.0 ± 4.5	10.9 ± 5.6	15.1 ± 15.1	10.3 ± 10.3	0.5 ± 0.5	0.0 ± 0.0
**H-bonds**							
**DRY - TM6**	R165^3.50^; T279^6.34^	0.0 ± 0.0	0.0 ± 0.0	0.0 ± 0.0	0.0 ± 0.0	0.0 ± 0.0	0.0 ± 0.0
**DRY - ICL2**	D164^3.49^; R179^ICL2^	99.6 ± 0.2	100.0 ± 0.0	99.7 ± 0.3	100 ± 0.0	97.5 ± 2.5	99.9 ± 0.1
**DRY - TM5**	R165^3.50^; Y252^5.58^	6.6 ± 3.3	1.9 ± 1.5	49.6 ± 23.7	1.7 ± 1.7	53.2 ± 11.7	33.3 ± 28.1
**CWxP - TM7**	C292^6.47^-W293^6.48^; N328^7.45^	0.5 ± 0.4	1.5 ± 1.5	0.1 ± 0.1	0.0 ± 0.0	18.8 ± 4.6	0.0 ± 0.0
**NPxxY- TM network**	N332^7.49^- Y336^7.53^; L158^3.43^- Y252^5.58^- V285^6.40^	13.1 ± 6.3	30.6 ± 7.5	1.4 ± 0.8	35.9 ± 19.3	6.6 ± 5.6	0.04 ± 0.4

* Ballesteros-Weinstein numbering is shown in superscript

Our simulation results supported the concept of an 'ionic lock' interaction between the DRY motif and TM6. This interaction was proposed to serve as a constraint in the inactive state, and its disruption upon receptor activation was suggested to facilitate the outward movement of TM6. This is an exclusive property for the inactive state since none of the active state MOP and its mutants showed the presence of the ‘ionic lock’ during the simulations. The identification of a distinctive salt bridge between R165^3.50^ of the DRY motif and D340^8.47^ of H8 was initially highlighted in our previous MD simulation study of the MOP [25]. However, in our current study, as well as in other previous studies of the β_2_AR and the CB1 receptors such interactions mostly remained absent, and could be deemed as transient, observed only in that particular case.

H-bonds were systematically present between D164^3.49^ of the DRY motif and R179 of the second intracellular loop (ICL2) in all MOP variants. This interaction was suggested previously to reorganize upon the activation of the β_2_AR [69], but that was not confirmed by our previous MD simulation studies of that particular receptor [26]. Here, minor structural destabilization of ICL2 of the Y326^7.43^F was found in one of the simulations, but it was not reproduced in the replicates. (Figure S6-S8) No clear correlation of this exception with the other analysis results is present that could justify the relevance of this observation. Furthermore, no clear trends could be identified for the frequencies of the CWxP-TM7 and NPxxY-TM network interactions. The DRY-TM5 interaction, proposed earlier to stabilize the G protein-bound active state was most frequently found in the N328^7.45^D mutant, which is in clear contradiction with the above presented results and inferences regarding the stability of this mutant receptor ligand complex and the integrity of its signaling state structure. These interaction patterns, observed originally in specific experimental GPCR structures, are not generally applicable as they were shown previously to differ largely in different GPCRs [25], [26], [27].

#### 3.2.4 Correlated dynamics among polar signaling channel residues

**Fig. 6:**
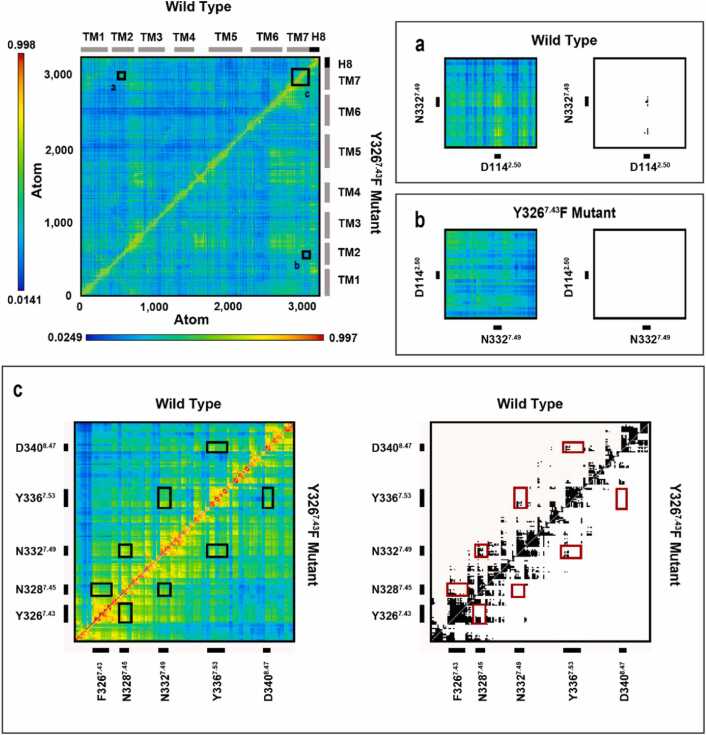
Generalized cross-correlation matrices of the active state, G_i_ protein-bound D340^8.47^N and D340^8.47^L MOP mutants. Panels (a-c) are magnified views of regions of amino acid residues of interest. Black and white panels show correlations above the threshold of 0.60 MI, which stands for moderate-to-high correlation.

**Fig. 7:**
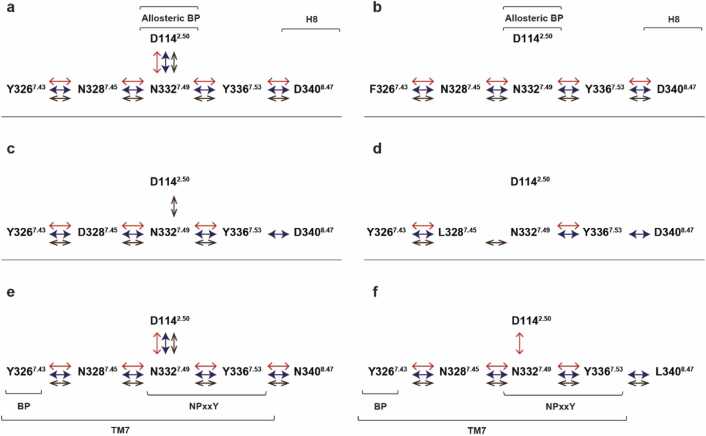
Correlated motions within the conserved polar signaling channel of active state, G_i_ protein-bound MOP derivatives revealed by generalized cross correlation matrix (GCCM) analysis. Arrows indicate correlated motions of respective amino acids. The color and shape of arrows correspond to the three replicate simulations of (a) wild type MOP and (b) Y326^7.43^F (c) N328^7.45^D (d) N328^7.45^L (e) D340^8.47^N (f) D340^8.47^L mutants.

The observation of the correlated motions among the polar signaling channel residues, indicated by GCCM analysis (Fig. 6, Figure S9-S10), has been consistent in the active, G protein bound state of the receptors, in our earlier as well as current studies. However, there are few differences among their dynamics which could be attributed to the specific properties of the mutant receptors. Fig. 7 shows the schematic representation of correlated motions observed between the channel residues of MOP and its mutants during the simulations. The complete correlation of movements of the polar signaling channel residues, pertaining to receptor activation and consequent signaling were reproduced in the wild type receptor system in both setups, as well as in the D340^8.47^N mutant simulation. Other mutant systems failed to show such absolute synchronous movement among the polar channel residues, suggesting that the only mutation that is possibly tolerated is the one which is distant from the orthosteric and allosteric binding pockets and in which the polarity is preserved. The involvement of D114^2.50^ in the correlated motions was particularly missing in these observations, underscoring the impact of D114^2.50^**-**N332^7.49^ internal dynamics in the activation and signaling of MOP [25], [33]. The ordered dynamics of the polar signaling channel, observed in the wild type and the D340^8.47^N mutant MOP receptors, may contribute to propagation of the activation signal in the form of charge perturbation towards the G protein coupling interface, assisted by water molecules present in the cavity. The presence of a hydrated pathway along the polar channel, with rapid exchange among the water molecules has been consistently observed in all relevant systems (Figure S11), and plays a role in signal transmission in the active receptor complexes [14], [70]. The results provide support to our hypothesis, that altering the polarity of the constituents of the signaling channel does affect the dynamics between the polar side chains, subsequently has a detrimental effect on signal transduction.

### 3.3 Pharmacological assessment

**Fig. 8:**
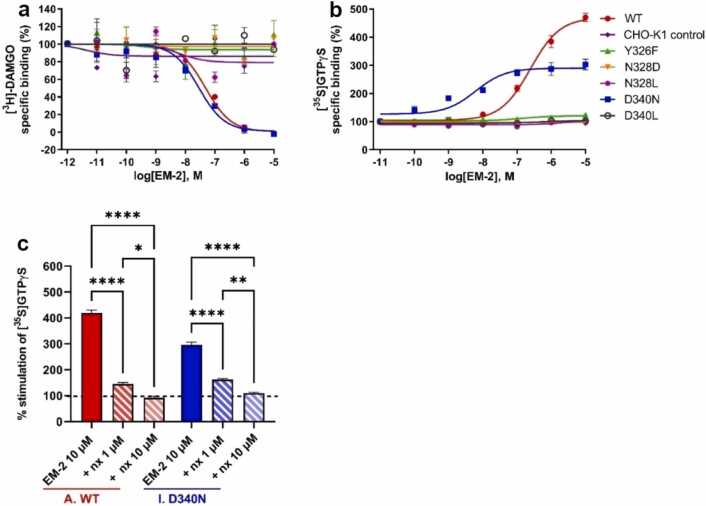
(a) MOP receptor binding affinity of EM2 in [^3^H]DAMGO competition binding assays to cell membrane homogenates. Figures represent the specific binding of the radioligand in percentage in the presence of increasing concentrations (10^−12^–10^−5^ M) of the indicated ligands. Data are expressed as percentage of mean specific binding ± S.E.M. (n ≥ 3). (b) G protein activation effects of EM2 ligand in [^35^S]GTPγS binding assays in cell membrane homogenates. Figures represent the relative specific binding of [^35^S]GTPγS in the presence of increasing concentrations (10^−11^–10^−5^ M) of the indicated compound, EM2. Data are expressed as percentage of mean specific binding ±S.E.M. (n ≥ 3) over the basal activity (100 %), (C) dose-dependent (1 and 10 µM) inhibition of 10 µM EM2 [^35^S]GTPγS binding by the MOP antagonist, naloxone (nx) in wild type and D340^8.47^N mutant cell membrane homogenates. 100 % is the basal activity. Data are expressed as a percentage of mean specific binding ± S.E.M. (n ≥ 3). ^****^, P < 0.0001, ^**^, P < 0.01, *, P < 0.05, one-way ANOVA followed by Tukey's multiple comparison test.

The results of *in vitro* radioligand competition binding experiments demonstrated, that EM2 was able to fully displace the radiolabeled prototypic agonist [^3^H]DAMGO from the orthosteric pocket the wild type MOP and the D340^8.47^N mutant. This was further corroborated by the comparable binding affinities, with an inhibition constant of 2.0 nM and 1.4 nM for the wild type and the mutant MOP, respectively, in good agreement with previously published data [59]. Conversely, no orthosteric interaction between EM2 and the rest of the mutant receptors was observed. (Fig. 8a, Table 3) Positive control binding experiments have been performed using unlabelled DAMGO, which provided corroborating results, confirming that the observed lack of binding in certain mutant constructs reflects genuine alterations in receptor-ligand interaction rather than methodological artifacts. (Figure S11)

**Table 3:** Binding affinity (K_i_) and signaling efficacy (E_max_) and potency (EC_50_) of the MOP selective endogenous peptide EM2 on membranes of CHO-K1 cells stably expressing the wild type and mutant MOP.

**Cells**	**EM2 vs [**^**3**^**H]-DAMGO**	**EM2 stimulated** **[**^**35**^**S]GTPγS binding**	
	**K**_**i**_**± S.E.M. (nM)**	**E**_**max**_**± S.E.M. (%)**	**EC**_**50**_**± S.E.M. (nM)**
**CHO-K1 MOP WT**	2 ± 0.3	437 ± 9.7^****^	245 ± 16***
**CHO-K1 negative control**	n.b.	94 ± 4.1	n.r
**CHO-K1 MOP Y326**^**7.43**^**F**	n.b.	113 ± 6.6	n.r
**CHO-K1 MOP N328**^**7.45**^**D**	n.b.	101 ± 2.8	n.r
**CHO-K1 MOP N328**^**7.45**^**L**	n.b.	107 ± 2.6	n.r
**CHO-K1 MOP D340**^**8.47**^**N**	1.4 ± 0.07	290 ± 9.8^****^	11 ± 4***
**CHO-K1 MOP D340**^**8.47**^**L**	n.b.	104 ± 2.8	n.r

EM-2: MOP agonist; n.b.: no binding. n.r.: not relevant n ≥ 3. Statistical comparison of stimulation efficacy (E_max_) was performed by one-way ANOVA followed by Tukey's multiple comparison test (^****^, P < 0.0001).

*** , significant difference (P < 0.05) from ligand potency (EC_50_) values were determined by unpaired Student's *t*-test, in the binding affinity (K_i_) values there was no significance.

When signaling potency of the wild type and mutant receptors was examined through agonist-stimulated [^35^S]GTPγS binding functional assays, it was observed that in both the wild type and the D340^8.47^N mutant MOP EM2 induced significant alterations in basal [^35^S]GTPγS binding which indicated G protein activation. (Fig. 7b, Table 3) Although EM2 exhibited a full agonistic effect on both receptors, there were notable differences in efficacy (E_max_) and potency (EC_50_) values. For the wild-type MOP, EM2 efficiently stimulated G proteins with high efficacy (437 %) and moderate potency (245 nM). In the D340^8.47^N mutant, EM2 demonstrated lower efficacy (290 %) but increased potency (11 nM). These variations in the ligand’s stimulatory effect suggest that the D340^8.47^N mutation does not diminish, yet affects receptor functionality. Consistent with the results of EM2 binding experiments, the other mutants failed to exhibit any functional activity. The agonist effect of EM2 was demonstrated to be dose-dependently inhibited by the clinically used MOP antagonist, naloxone, in both wild type and D340^8.47^N mutant receptors. The results indicated that nearly all mutant systems led to the complete loss of both ligand binding and signaling capability. D340^8.47^N as mentioned, preserved or maintained the polarity in the channel. This observation perfectly corroborated our findings from simulation studies, where this remained the only mutant showing complete correlated motions among the polar side chains. Another particularly significant observation was that a radical mutation in the intracellular G protein coupling region (D340^8.47^L) resulted in impaired ligand binding at the orthosteric region (Fig. 1, Fig. 7, Table 3). This outcome provides direct support to the hypothesis of a strong allosteric linkage between these two sites crucial for receptor activation.

### 3.4 Assessment of mutation effects on ligand binding via MMPBSA calculations

The inclusion of the entropic term in MMPBSA calculations introduced large errors, which made the calculated binding free energies (ΔG) unreliable. Therefore, only the enthalpy change (ΔH) was considered for the estimation of the strength of association of EM2 and the wild type and mutant MOP receptors. The results of MMPBSA calculations indicated stronger interactions of EM2 with the wild type receptor and the D340N and N328L mutants (Table 4). The differences of ΔH are not as striking as the differences found in *in vitro* experiments. However, even a very small, less than 1.0 kcal mol^−1^ difference in binding free energy can result in an order of magnitude difference in receptor affinity expressed by inhibition constants (K_i_). The results obtained for the receptors are in agreement with the experimental binding data, except for the N328L mutant. Further investigation is needed for the adequate explanation of this false positive result.

**Table 4:** Strength of intramolecular EM2-MOP interaction calculated by MMPBSA.

	**ΔH / kcal mol**^**−1**^	**ΔH-TΔS / kcal mol**^**−1**^
**WT**	−49.55 ± 0.31	−22.03 ± 3.61
**Y326F**	−43.62 ± 2.16	3.42 ± 9.92
**N328D**	−47.39 ± 1.75	−16.51 ± 4.86
**N328L**	−51.08 ± 0.61	−32.99 ± 3.41
**D340N**	−53.88 ± 2.39	−31.46 ± 13.24
**D340L**	−47.99 ± 0.93	−26,43 ± 3.43

## 4 Conclusions

The existence of interconnecting microswitches extending from the ligand binding pocket to the intracellular surface, and its prime importance in the activation and signaling of class A GPCRs has been well established already in experiments as well as computational studies [15], [20]. However, most of the discourse was based on large-scale structural rearrangements of the TM domain and associated macroscopic changes. These also give in to the bottleneck formed in situations, when multiple active states of the receptor or structurally analogous but functionally different ligands are considered and no sufficiently accurate explanation for the activation of different signaling pathways could be given based on structural rearrangements alone. As proposed earlier, an additional event underlying these phenomena could be a shift in the charge balance and the consequent polarization of the receptor. Such polarization could be contributed by the concerted motions of polar species in the TM domain, as well as the nature of the orthosteric ligand [25], [26], [27]. GCCM analysis confirmed such concerted motions in the polar signaling channel in the wild type, as well as the D340^8.47^N mutant receptors, but not in the other mutants, indicating that significant alteration of the polarity of channel constituents may alter the ligand binding and the signaling capacity of the MOP. The bioactive orientation of the ligand and its native free dynamics in the orthosteric pocket was also suggested to be essential for receptor activation. The conclusions drawn from the simulation data were corroborated by results of *in vitro* competitive radioligand binding assays as well as [^35^S]GTPγS binding functional assays. Furthermore, experimental results confirmed strong coupling between the orthosteric binding pocket and the intracellular surface of the MOP. The results obtained from our current study along with earlier findings provide further insights into the activation pathways of class A GPCRs which could have important implications for the development of new therapeutic strategies targeting this receptor family. More specifically, the new perspective introduced, concerning the electrostatic balance of the TM domain could open new avenues in the development of novel, pathway specific drugs.

## CRediT authorship contribution statement

**Szabolcs Dvorácskó:** Writing – review & editing, Methodology, Investigation, Formal analysis. **Arijit Sarkar:** Writing – review & editing, Writing – original draft, Visualization, Methodology, Investigation, Formal analysis. **Argha Mitra:** Methodology. **Zoltán Lipinszki:** Writing – review & editing, Methodology. **Krisztina Buzás:** Writing – review & editing. **Mária Harmati:** Writing – review & editing, Methodology. **Attila Borics:** Writing – review & editing, Writing – original draft, Visualization, Supervision, Project administration, Methodology, Conceptualization.

## Informed consent statement

Not applicable.

## Institutional review board statement

Not applicable.

## Funding

This research was funded by the OTKA-K143124 grant, awarded to A.B. by the National Research, Development and Innovation Office. Scholarship for A.S. was provided by the ‘Stipendium Hungaricum’ program of the Hungarian Ministry of Foreign Affairs and Trade and the Tempus Public Foundation (SH identifier: 103685). D.Sz. was supported by the grant OTKA-PD139012 of the National Research Development and Innovation Ofﬁce and the János Bolyai Research Scholarship of the Hungarian Academy of Sciences. Z.L received support from the National Laboratory for Biotechnology (2022-2.1.1-NL-2022-00008) and the 10.13039/501100003825Hungarian Academy of Sciences (Lendület Program Grant (LP2017-7/2017). K. B. received funding from TKP2021-EGA-09 and OTKA-K143255 grants provided by the National Research, Development and Innovation Office.

## Declaration of Competing Interest

The authors declare the following financial interests/personal relationships which may be considered as potential competing interests: Attila Borics reports financial support was provided by National Research Development and Innovation Office. Szabolcs Dvorácskó reports financial support was provided by National Research Development and Innovation Office. Arijit Sarkar reports financial support was provided by Hungarian Ministry of Foreign Affairs and Trade. Szabolcs Dvorácskó reports financial support was provided by Hungarian Academy of Sciences. Zoltan Lipinszki reports financial support was provided by Hungarian Academy of Sciences. Zoltán Lipinszki reports financial support was provided by National Laboratory for Biotechnology. Krisztina Buzás reports financial support was provided by National Research Development and Innovation Office. If there are other authors, they declare that they have no known competing financial interests or personal relationships that could have appeared to influence the work reported in this paper.

## Data Availability

All data presented in the article and the Supplementary Materials are available upon request from the corresponding author.
