# Supplementary material for: Evidencing the role of a conserved polar signaling channel in the activation mechanism of the μ-opioid receptor
Source: Comput Struct Biotechnol J. 2025 Jul 16;27:3216–28. doi: 10.1016/j.csbj.2025.07.014 (PMC12309854; doi:10.1016/j.csbj.2025.07.014)
Supplement: Supplementary file 1 — Supplementary material [file mmc1.docx]

**SUPPLEMENTARY MATERIAL**

for

**Evidencing the role of a conserved polar signaling channel in the activation mechanism of the μ-opioid receptor**

**Arijit Sarkar^a,b^, Szabolcs Dvorácskó^a^_,_ Zoltán Lipinszki^c,d^_,_ Argha Mitra^a^, Mária Harmati^e,f^, Krisztina Buzás^e,f^, Attila Borics^a,*^**

^a^ Laboratory of Biomolecular Structure and Pharmacology, Institute of Biochemistry, HUN-REN Biological Research Centre, Szeged, Hungary

^b^ Theoretical Medicine Doctoral School, Albert Szent-Györgyi Medical School, University of Szeged, Hungary

^c^ Synthetic and Systems Biology Unit, Institute of Biochemistry, HUN-REN Biological Research Centre, Szeged, Hungary

^d^ National Laboratory for Biotechnology, Institute of Genetics, HUN-REN Biological Research Centre, Szeged, Hungary

^e^ Laboratory of Microscopic Image Analysis and Machine Learning, Institute of Biochemistry, HUN-REN Biological Research Centre, Szeged, Hungary

^f^ Department of Immunology, Albert Szent-Györgyi Medical School, Faculty of Science and Informatics, University of Szeged

* corresponding author, lead contact

**Keywords:** G protein-coupled receptors, opioid, molecular dynamics, activation mechanism, signal transduction, polar signaling channel, mutation, pharmacological assessment

**Table S1.** Post-translational modifications of MOP included in the simulation systems

| **Glycosylation** | **Phosphorylation** | **Lipidation / Palmitoylation** |
| --- | --- | --- |
| N9, N31, N38 (a) | S363, T370 (b) | C170^3.55^ (c) |

1. Huang P, Chen C, Xu W, Yoon SI, Unterwald EM, *et al*.. *Biochemical and Biophysical Research Communications* **365**, 82-88 (2008). DOI:[10.1016/j.bbrc.2007.10.128](https://pubmed.ncbi.nlm.nih.gov/17980152/)
2. Mann A, Illing S, Miess E, Schulz S. *British Journal of Pharmacology* **172**, 311-316 (2015). DOI:[10.1111/bph.12627](https://bpspubs.onlinelibrary.wiley.com/doi/full/10.1111/bph.12627)
3. Zheng H, Pearsall EA, Hurst DP, Zhang Y, Chu J, *et al*. *BMC Cell Biology* **13**, 1-18 ( 2012). DOI:[10.1186/1471-2121-13-6](https://bmcmolcellbiol.biomedcentral.com/articles/10.1186/1471-2121-13-6)

**Table S2.** Oligonucleotide primers used in the study

| **Primer name** | **Sequence (5’-3’)** | **Used for** |
| --- | --- | --- |
| T7 promoter forward | TAATACGACTCACTATAGGG | Cloning MOP-1 into pJET2.1 and for sequencing |
| BGH reverse | TAGAAGGCACAGTCGAGG |  |
| MOP_Y326F_fw | TGCATTGCCTTGGGTTTCACAAACAGCTGCCTG | *In vitro* mutagenesis |
| MOP_Y326F_rev | CAGGCAGCTGTTTGTGAAACCCAAGGCAATGCA |  |
| MOP_N328D_fw | GCCTTGGGTTACACAGACAGCTGCCTGAACCCA |  |
| MOP_N328D_rev | TGGGTTCAGGCAGCTGTCTGTGTAACCCAAGGC |  |
| MOP_N328L_fw | GCCTTGGGTTACACACTCAGCTGCCTGAACCCA |  |
| MOP_N328L_rev | TGGGTTCAGGCAGCTGAGTGTGTAACCCAAGGC |  |
| MOP_D340N_fw | CTTTATGCGTTCCTGAATGAAAACTTCAAACGA |  |
| MOP_D340N_rev | TCGTTTGAAGTTTTCATTCAGGAACGCATAAAG |  |
| MOP_D340L_fw | CTTTATGCGTTCCTGCTTGAAAACTTCAAACGA |  |
| MOP_D340L_rev | TCGTTTGAAGTTTTCAAGCAGGAACGCATAAAG |  |
| InFusion MOP fw | CGGTACCCGGGGATCGAATTCGCCCTTGAGAGGAAGAGG | In-fusion cloning to pTRE2hyg |
| InFusion MOP rev | GCTGACTAGAGGATCGAATTCGCCCTTCAGGAAACC |  |
| pTre2Hyg fw | ACGCTGTTTTGACCTCCATAG | Sequencing |
| pTre2Hyg rev | ATGAATTTTACAATAGCGAA |  |

**Table S3.** χ^1^ Rotamer populations of aromatic amino acid side chains of EM2 during simulations when bound to MOP derivatives. Values represent mean ± SEM from three independent replicates (n = 3).

| **Tyr^1^** | ***g^+^*** | ***g^-^*** | ***t*** |
| --- | --- | --- | --- |
| WT | 0.00 ± 0.00 | 0.03 ± 0.03 | 0.80 ± 0.14 |
| Y326^7.43^F* | 0.00 ± 0.00 | 0.00 ± 0.00 | 0.95 ± 0.02 |
| N328^7.45^D | 0.00 ± 0.00 | 0.00 ± 0.00 | 0.94 ± 0.02 |
| N328^7.45^L | 0.00 ± 0.00 | 0.00 ± 0.00 | 0.95 ± 0.03 |
| D340^8.47^N | 0.00 ± 0.00 | 0.00 ± 0.00 | 0.93 ± 0.02 |
| D340^8.47^L | 0.00 ± 0.00 | 0.00 ± 0.00 | 0.98 ± 0.01 |
| **Phe^3^** |  |  |  |
| WT | 0.00 ± 0.00 | 0.83 ± 0.16 | 0.16 ± 0.16 |
| Y326^7.43^F | 0.00 ± 0.00 | 0.40 ± 0.30 | 0.59 ± 0.30 |
| N328^7.45^D | 0.00 ± 0.00 | 0.68 ± 0.32 | 0.32 ± 0.32 |
| N328^7.45^L | 0.00 ± 0.00 | 0.67 ± 0.30 | 0.33 ± 0.30 |
| D340^8.47^N | 0.00 ± 0.00 | 0.93 ± 0.07 | 0.07 ± 0.07 |
| D340^8.47^L | 0.00 ± 0.00 | 0.65 ± 0.32 | 0.35 ± 0.32 |
| **Phe^4^** |  |  |  |
| WT | 0.01 ± 0.01 | 0.99 ± 0.01 | 0.00 ± 0.00 |
| Y326^7.43^F | 0.00 ± 0.00 | 0.99 ± 0.00 | 0.00 ± 0.00 |
| N328^7.45^D | 0.00 ± 0.00 | 0.99 ± 0.00 | 0.00 ± 0.00 |
| N328^7.45^L | 0.11 ± 0.11 | 0.89 ± 0.11 | 0.00 ± 0.00 |
| D340^8.47^N | 0.00 ± 0.00 | 1.00 ± 0.00 | 0.00 ± 0.00 |
| D340^8.47^L | 0.00 ± 0.00 | 0.99 ± 0.01 | 0.01 ± 0.01 |

* Ballesteros-Weinstein numbering is shown in superscript

**Table S4.** Results of clustering

|  | **replica 1** | | | | | | |
| --- | --- | --- | --- | --- | --- | --- | --- |
|  | **1.0 Å^a^** | | |  | **1.5 Å^a^** | | |
|  | **N_cl_^b^** | **N_cl1%_^c^** | **ΣP(cl>1%)^d^ / %** |  | **N_cl_^b^** | **N_cl1%_^c^** | **ΣP(cl>1%)^d^ / %** |
| **WT** | 40 | 10 | 93.4 |  | 5 | 3 | 99.0 |
| **Y326F** | 51 | 14 | 91.4 |  | 8 | 4 | 98.9 |
| **N328D** | 98 | 26 | 79.9 |  | 13 | 6 | 98.6 |
| **N328L** | 66 | 20 | 91.2 |  | 7 | 3 | 98.3 |
| **D340N** | 43 | 14 | 94.8 |  | 6 | 3 | 99.6 |
| **D340L** | 43 | 11 | 91.8 |  | 5 | 2 | 99.6 |

|  | **replica 2** | | | | | | |
| --- | --- | --- | --- | --- | --- | --- | --- |
|  | **1.0 Å^a^** | | |  | **1.5 Å^a^** | | |
|  | **N_cl_^b^** | **N_cl1%_^c^** | **ΣP(cl>1%)^d^ / %** |  | **N_cl_^b^** | **N_cl1%_^c^** | **ΣP(cl>1%)^d^ / %** |
| **WT** | 39 | 11 | 94.8 |  | 5 | 3 | 99.6 |
| **Y326F** | 63 | 15 | 87.5 |  | 6 | 3 | 98.7 |
| **N328D** | 63 | 18 | 90.0 |  | 8 | 4 | 99.4 |
| **N328L** | 46 | 14 | 94.5 |  | 7 | 4 | 99.7 |
| **D340N** | 43 | 11 | 93.1 |  | 4 | 2 | 99.8 |
| **D340L** | 58 | 17 | 91.3 |  | 8 | 3 | 99.1 |

|  | **replica 3** | | | | | | |
| --- | --- | --- | --- | --- | --- | --- | --- |
|  | **1.0 Å^a^** | | |  | **1.5 Å^a^** | | |
|  | **N_cl_^b^** | **N_cl1%_^c^** | **ΣP(cl>1%)^d^ / %** |  | **N_cl_^b^** | **N_cl1%_^c^** | **ΣP(cl>1%)^d^ / %** |
| **WT** | 51 | 13 | 91.6 |  | 7 | 3 | 98.9 |
| **Y326F** | 50 | 16 | 92.1 |  | 5 | 2 | 98.8 |
| **N328D** | 62 | 19 | 86.3 |  | 6 | 4 | 99.6 |
| **N328L** | 43 | 14 | 94.8 |  | 5 | 2 | 98.9 |
| **D340N** | 36 | 12 | 94.8 |  | 5 | 3 | 99.1 |
| **D340L** | 56 | 12 | 89.6 |  | 7 | 4 | 99.3 |

^a^ Backbone RMSD similarity cutoff.
^b^ Number of clusters found at the respective similarity cutoff.

^c^ Number of clusters found at the respective similarity cutoff which had a population higher than 1% of

the total structural ensemble.

^d^ The sum of population of the clusters with population higher than 1% of the total structural ensemble.


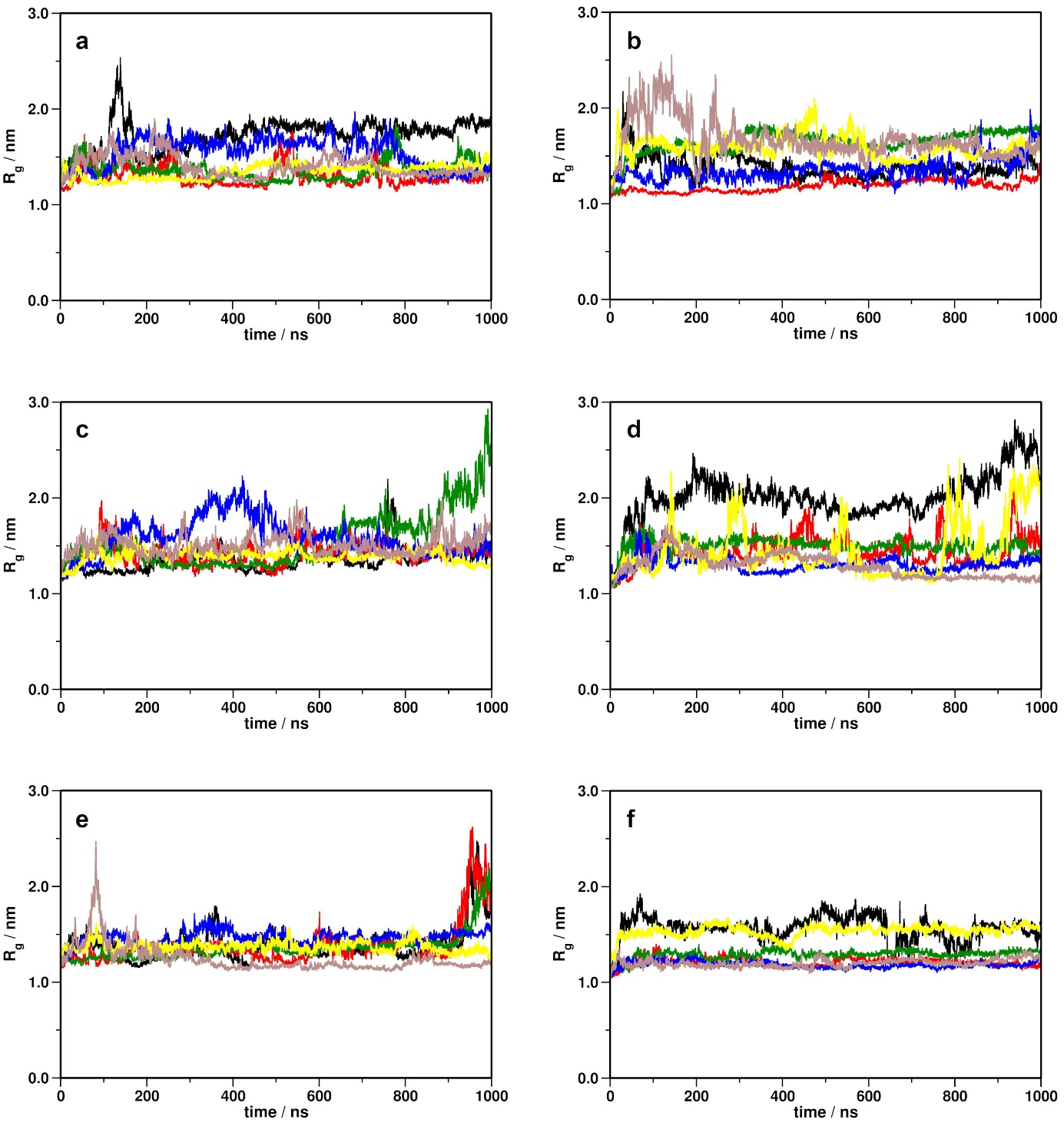


**Figure S1.** Radii of gyration of the N- (a, c, e) and C-terminal (b, d, f) domains during the first (a, b), second (c, d) and third (e, f) set of simulations**.** Black: wild type MOP; red: Y326^7.43^F; green: N328^7.45^D; blue: N328^7.45^L; orange: D340^8.47^N; brown: D340^8.47^L.


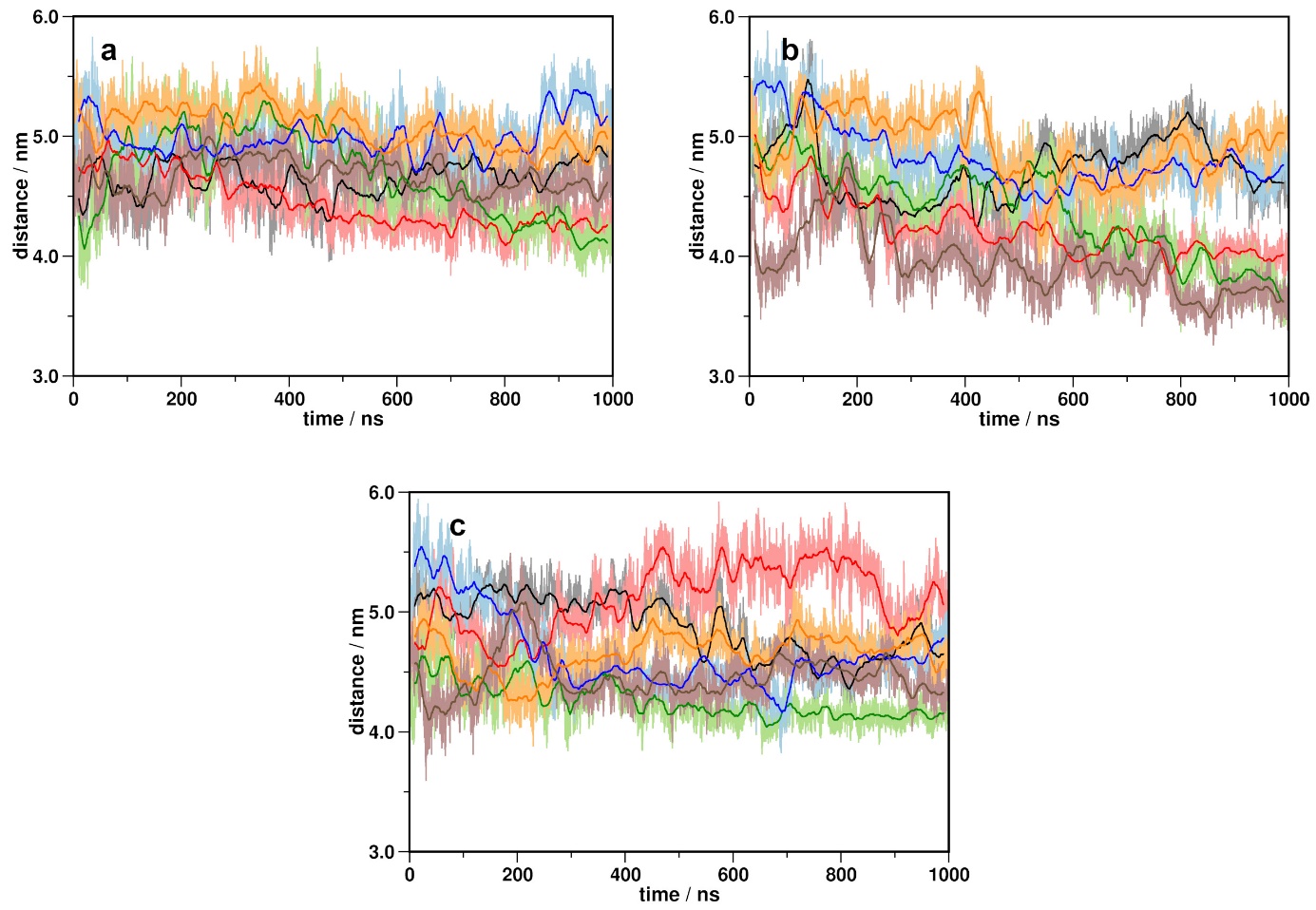


**Figure S2.** Minimum distance between the N- and C-terminal domains during the first (a), second (b) and third (c) set of simulations**.** Black: wild type MOP; red: Y326^7.43^F; green: N328^7.45^D; blue: N328^7.45^L; orange: D340^8.47^N; brown: D340^8.47^L.


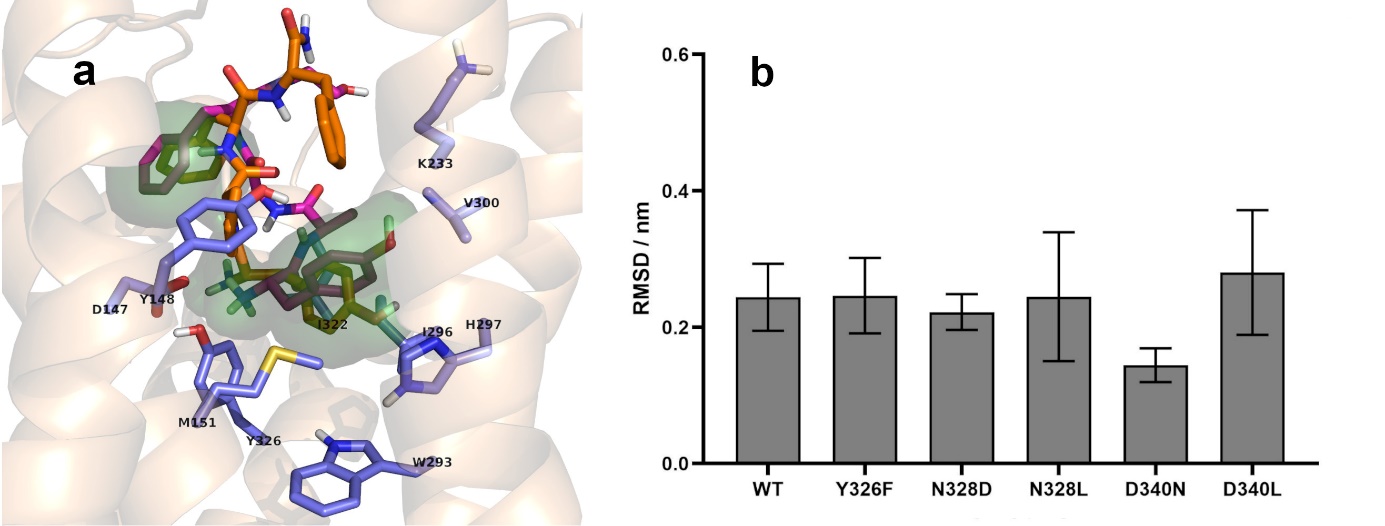


**Figure S3.** (a) Bioactive orientation of EM2 (orange) in the binding pocket of MOP, superimposed with the cryo-EM structure of DAMGO (magenta, PDB ID: 6DDE). Overlapping pharmacophore groups of the two ligands are highlighted with green. (b) Disposition of EM2 from its initial position during the course of simulations. Bars represent the mean ± SEM of three independent replicates (n = 3). Statistical significance was assessed using one-way ANOVA followed by Tukey’s multiple comparisons test. No statistically significant differences were observed among the groups (p > 0.05).


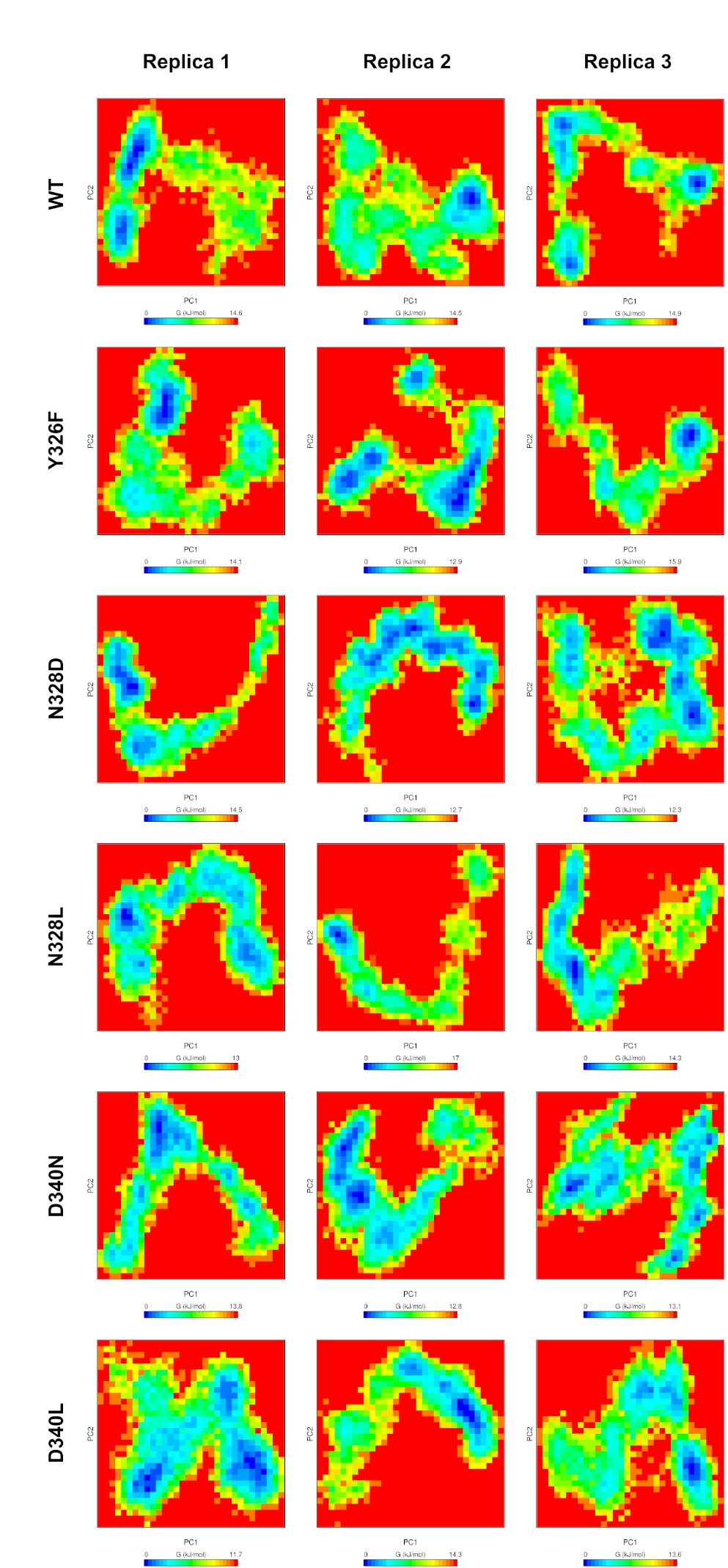


**Figure S4.** Gibbs free energy landscapes of all conducted simulations


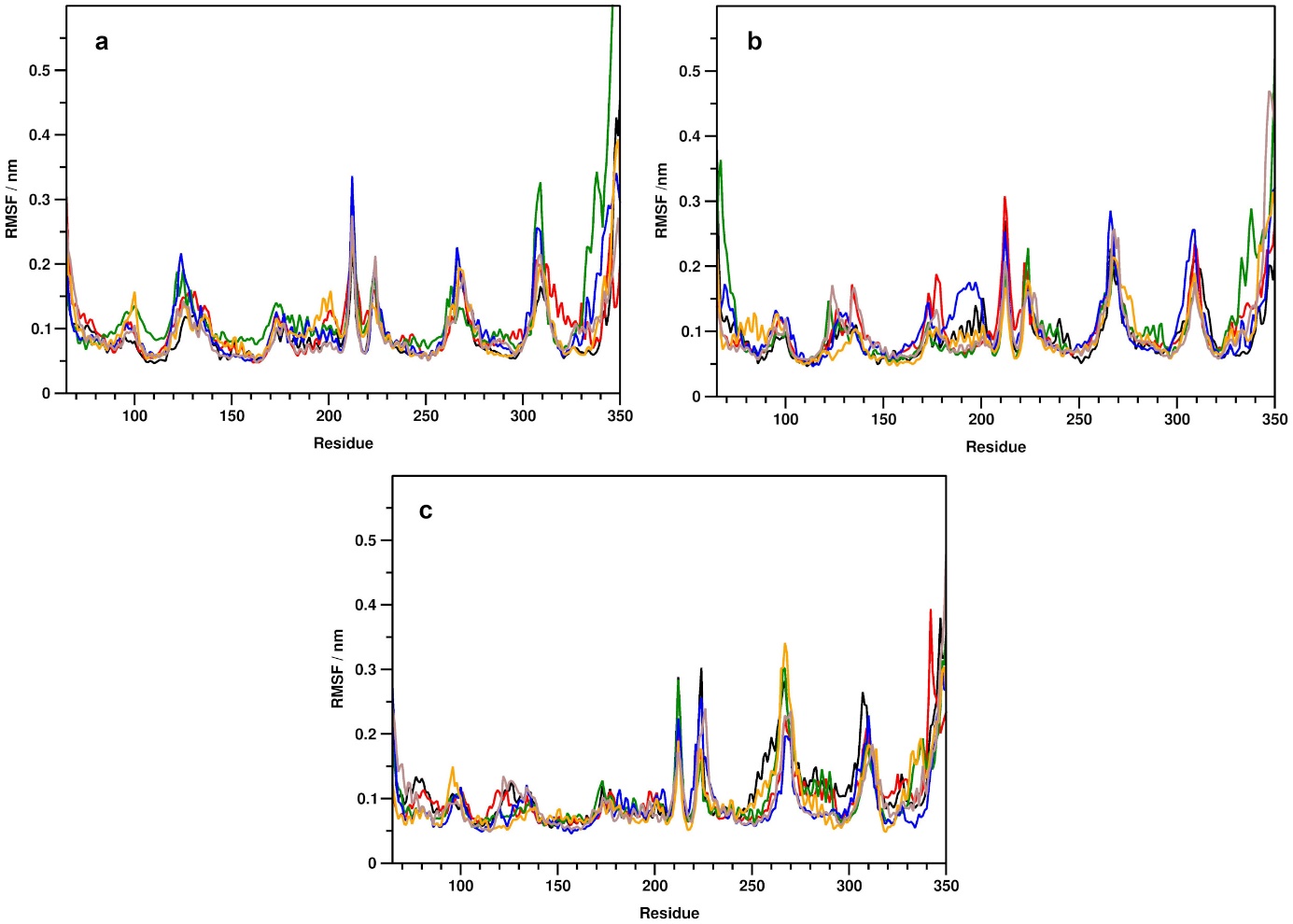


**Figure S5.** Root mean square fluctuations of TM residues in the first (a), second (b) and third (c) set of simulations. Black: wild type MOP; red: Y326^7.43^F; green: N328^7.45^D; blue: N328^7.45^L; orange: D340^8.47^N; brown: D340^8.47^L.

**
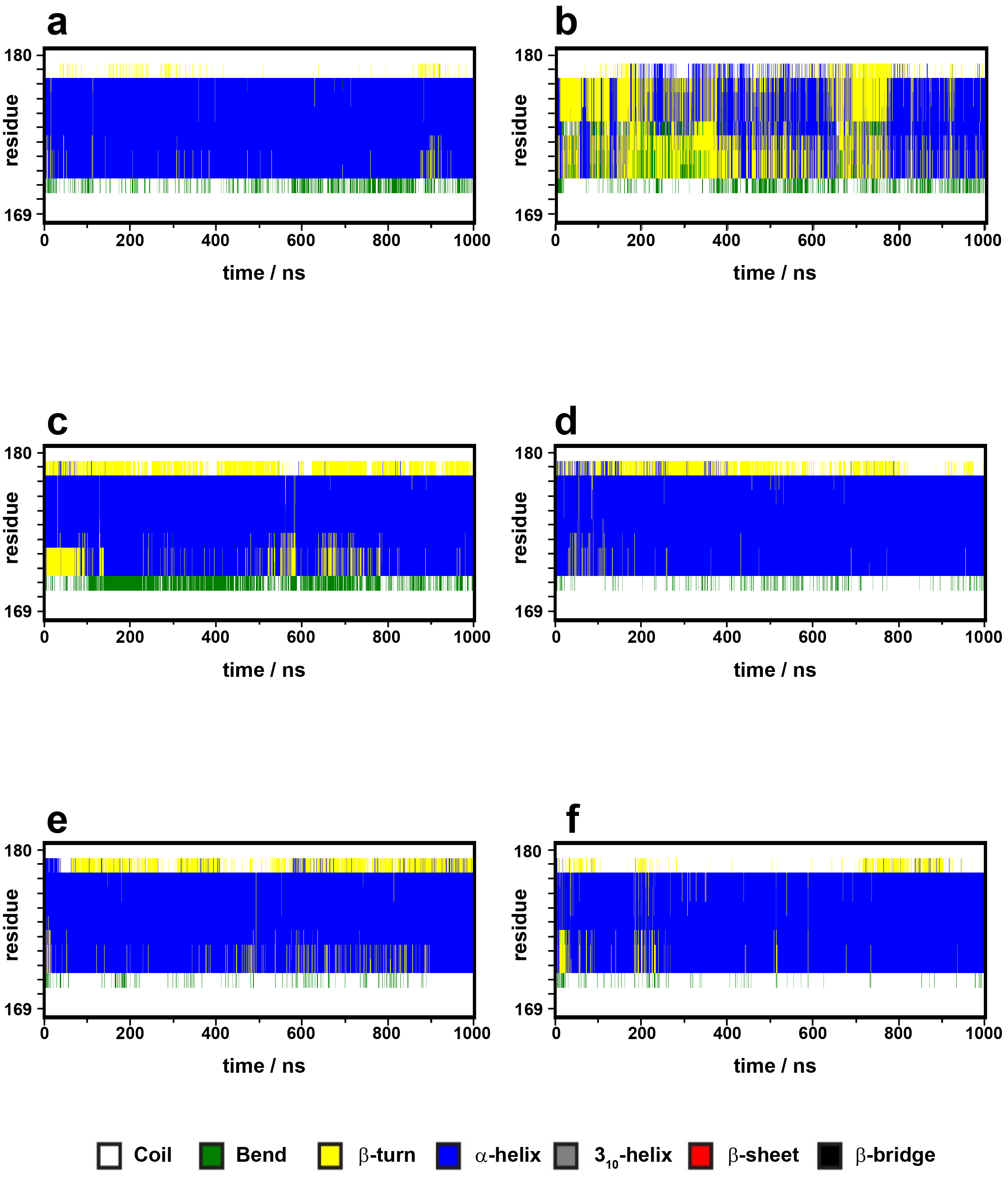
**

**Figure S6.** Evolution of the secondary structure of ICL2 during the first set of simulations. (a) wild type MOP (b) Y326^7.43^F (c) N328^7.45^D (d) N328^7.45^L (e) D340^8.47^N (f) D340^8.47^L.


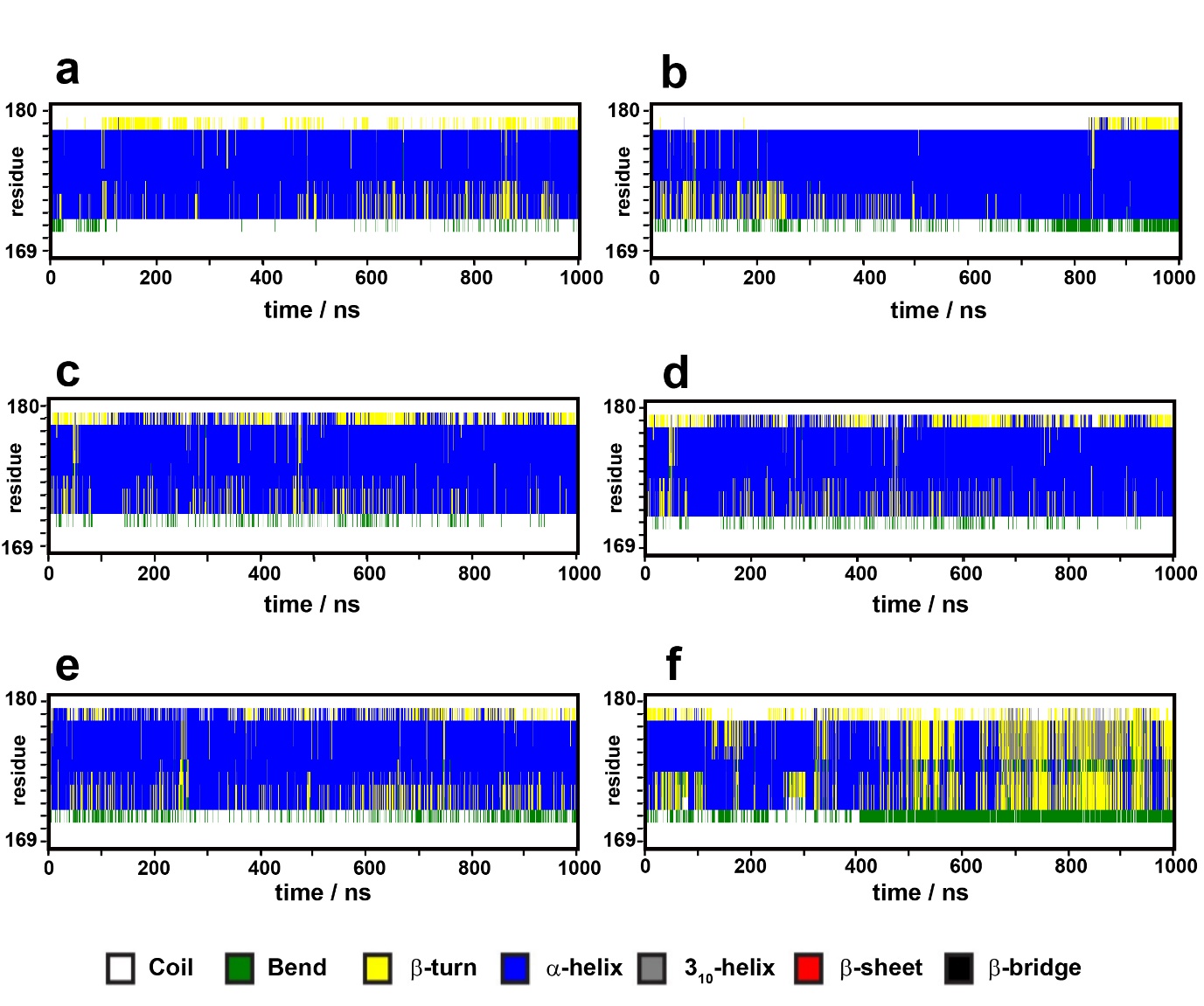


**Figure S7.** Evolution of the secondary structure of ICL2 during the second set of simulations. (a) wild type MOP (b) Y326^7.43^F (c) N328^7.45^D (d) N328^7.45^L (e) D340^8.47^N (f) D340^8.47^L.


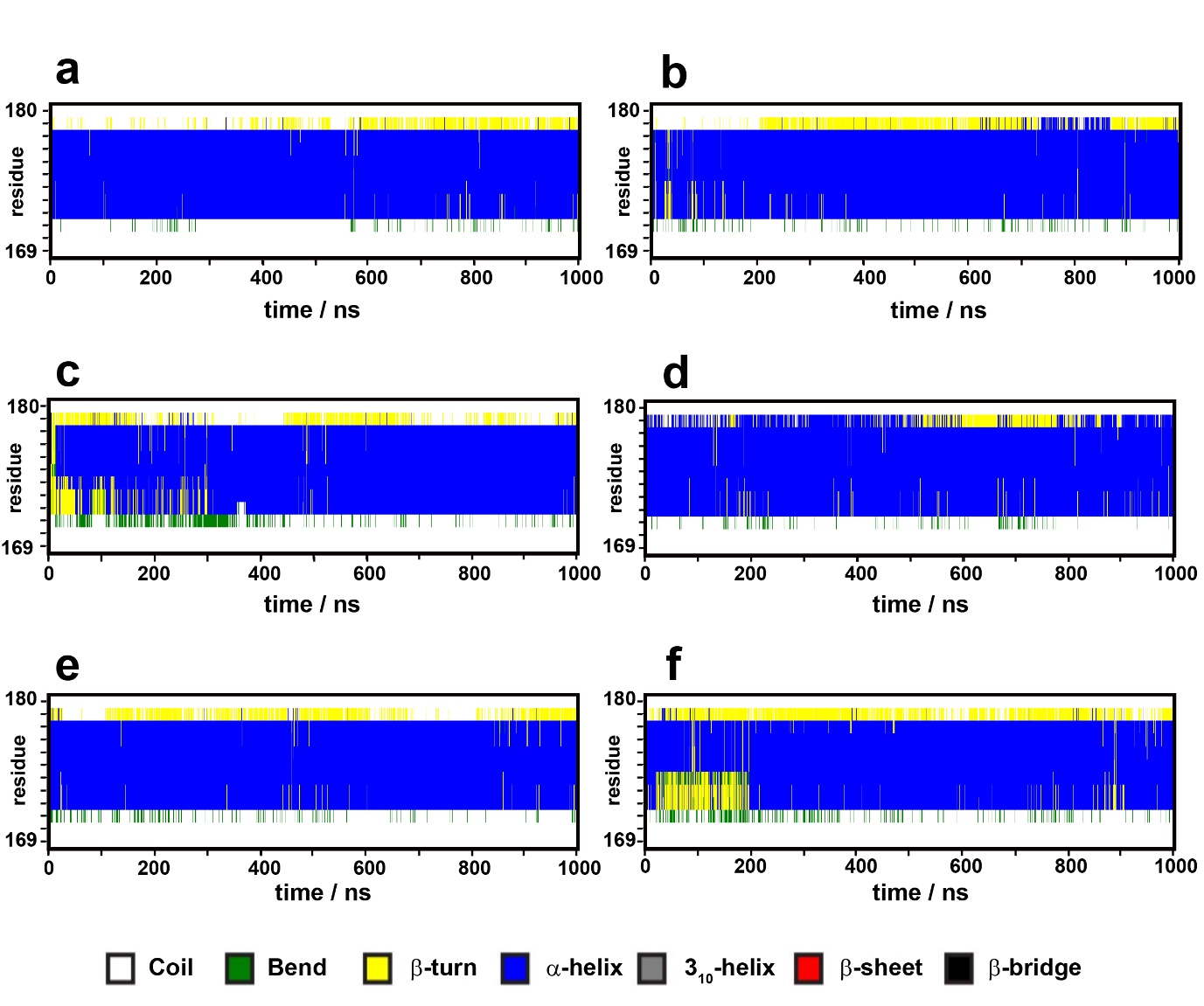


**Figure S8.** Evolution of the secondary structure of ICL2 during the third set of simulations. (a) wild type MOP (b) Y326^7.43^F (c) N328^7.45^D (d) N328^7.45^L (e) D340^8.47^N (f) D340^8.47^L.

**
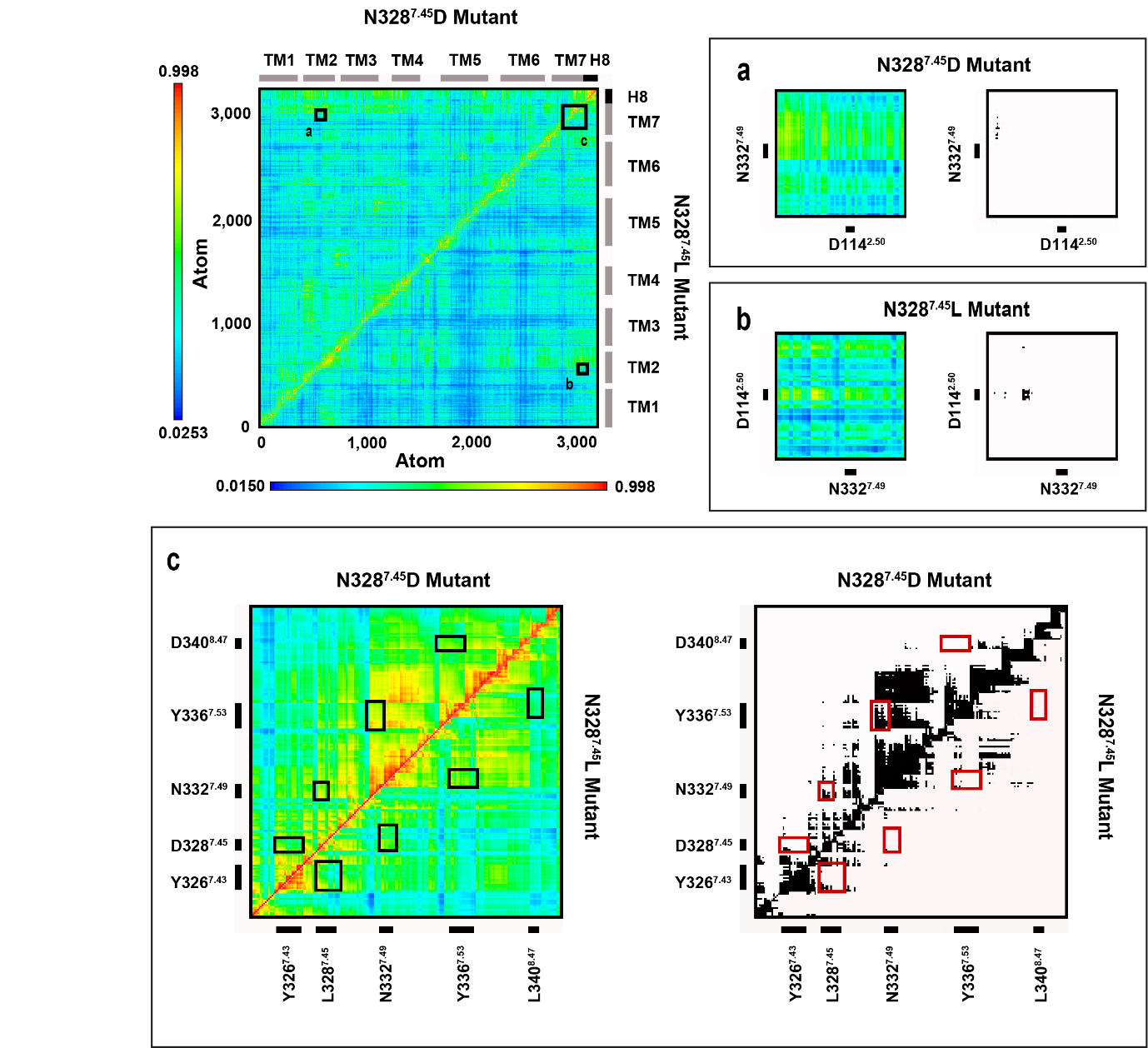
**

**Figure S9.** Generalized cross-correlation matrices of the acive state, G_i_ protein-bound N328^7.45^D and N328^7.45^L MOP mutants. Panels (a-c) are magnified views of regions of amino acid residues of interest. Black and white panels show correlations above the threshold of 0.60 MI, which stands for moderate-to-high correlation.

**
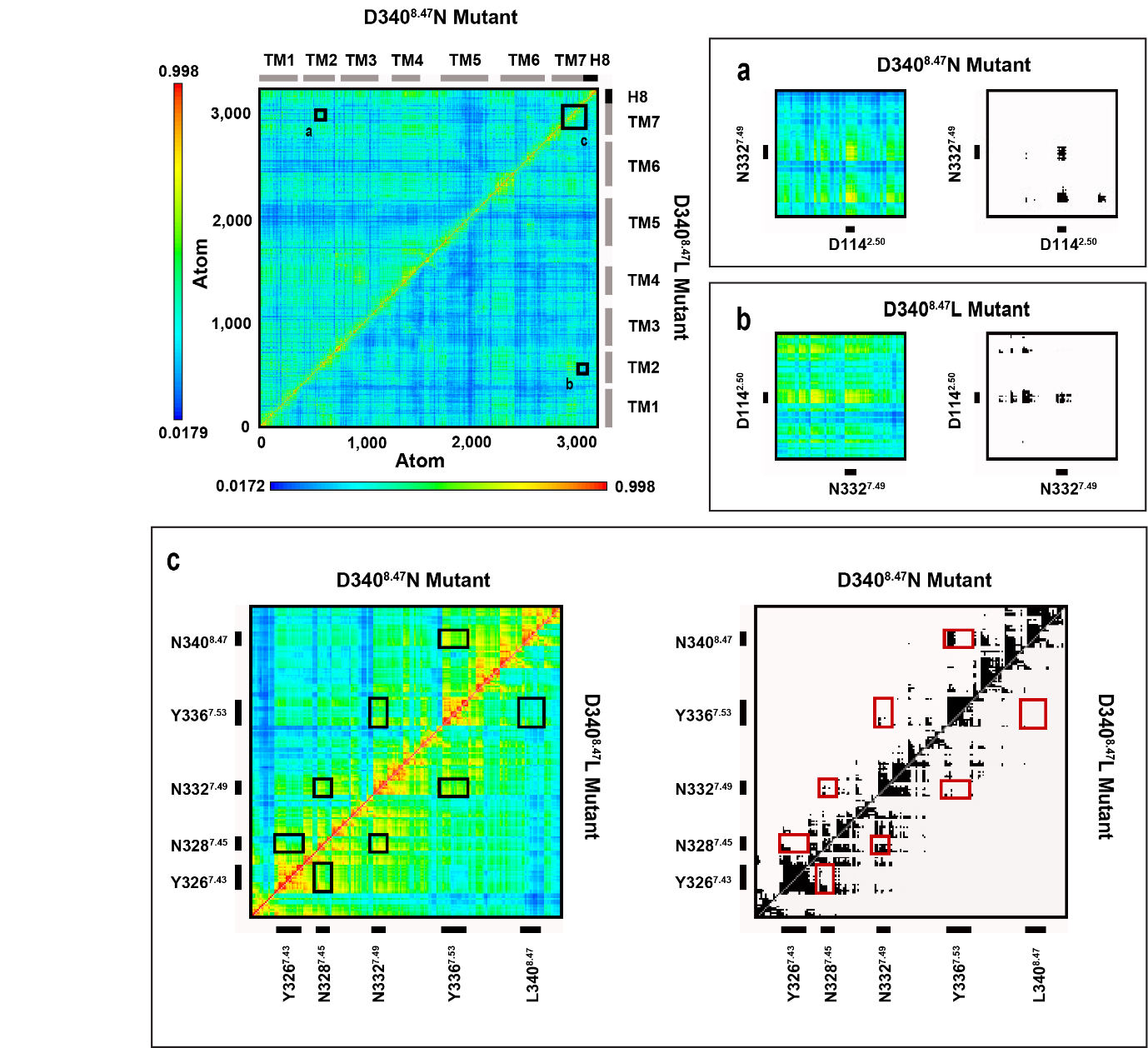
**

**Figure S10.** Generalized cross-correlation matrices of the active state, G_i_ protein-bound D340^8.47^N and D340^8.47^L MOP mutants. Panels (a-c) are magnified views of regions of amino acid residues of interest. Black and white panels show correlations above the threshold of 0.60 MI, which stands for moderate-to-high correlation.

**
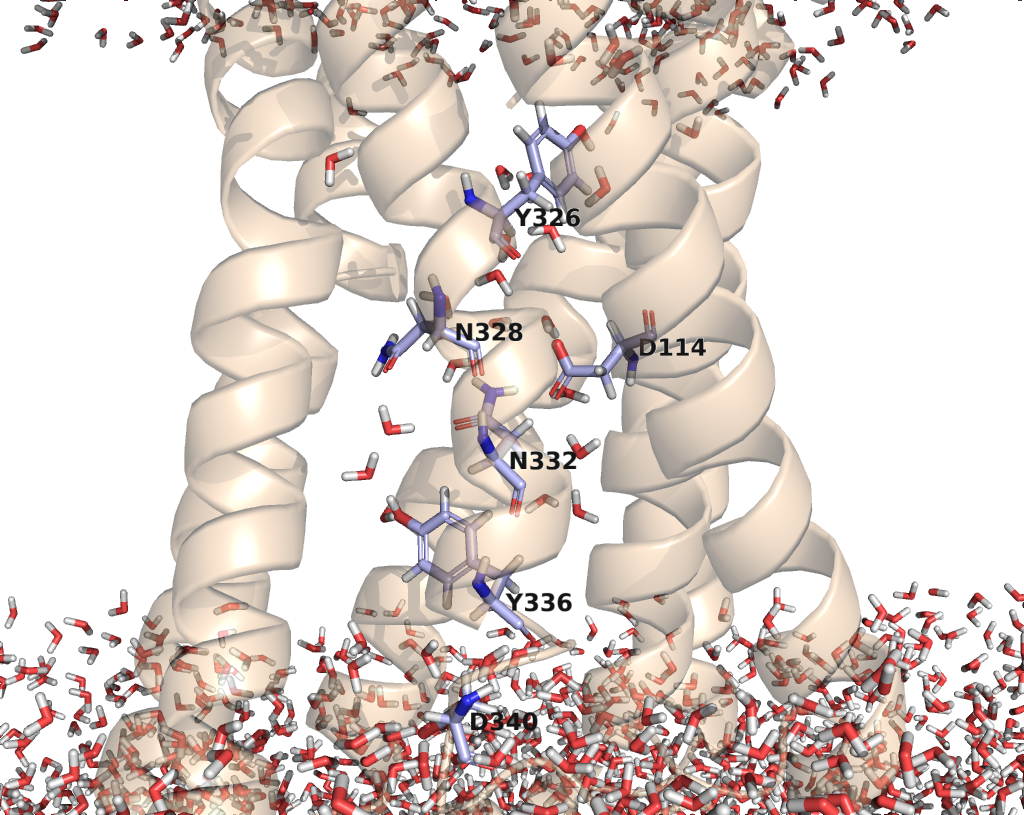
Figure S11.** Snapshot of the transmembrane domain of the active, EM2-bound, G_i_ protein-coupled wild type MOP. Water molecules and polar signaling channel residues in contact with water during the course of simulation are shown in stick representation.


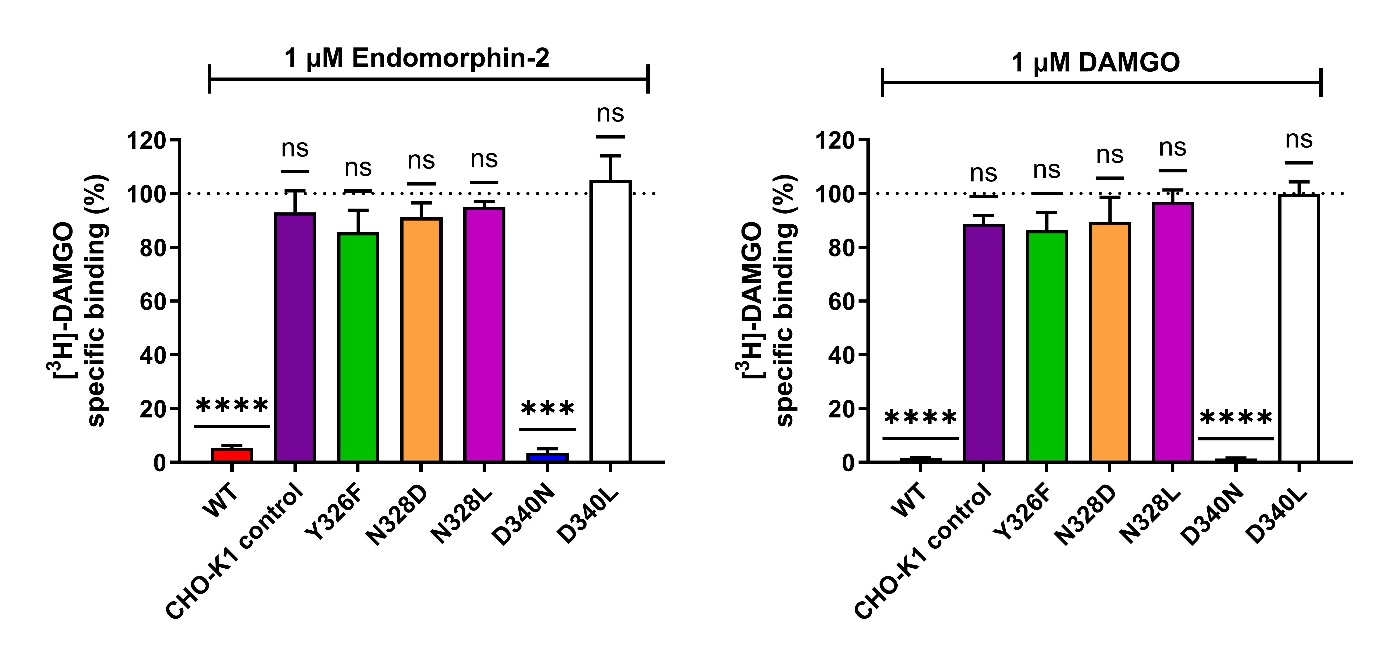


**Figure S12.** MOP receptor binding affinity of Endomorphin-2 and DAMGO was evaluated using [³H]DAMGO competition binding assays performed on cell membrane homogenates. The figure presents the percentage of specific [³H]DAMGO binding in the presence of 1 µM concentrations of the indicated ligands. Data are expressed as percentage of mean specific binding ± S.E.M. (n ≥ 3). Statistical analysis was performed using a one-sample t-test compared to the 100% reference; **** P < 0.0001, *** P < 0.001; ns: not significant.
